# Paradoxical myeloid-derived suppressor cell reduction in the bone marrow of SIV chronically infected macaques

**DOI:** 10.1371/journal.ppat.1006395

**Published:** 2017-05-12

**Authors:** Yongjun Sui, Blake Frey, Yichuan Wang, Rolf Billeskov, Shweta Kulkarni, Katherine McKinnon, Tracy Rourke, Linda Fritts, Christopher J. Miller, Jay A. Berzofsky

**Affiliations:** 1 Vaccine Branch, National Cancer Institute, National Institutes of Health, Bethesda, MD, United States of America; 2 Center for Comparative Medicine, University of California Davis, Davis, CA, United States of America; Emory University, UNITED STATES

## Abstract

Myeloid derived suppressor cells (MDSCs), which suppress anti-tumor or anti-viral immune responses, are expanded in the peripheral blood and tissues of patients/animals with cancer or viral infectious diseases. We here show that in chronic SIV infection of Indian rhesus macaques, the frequency of MDSCs in the bone marrow (BM) was paradoxically and unexpectedly decreased, but increased in peripheral blood. Reduction of BM MDSCs was found in both CD14^+^MDSC and Lin^-^CD15^+^MDSC subsets. The reduction of MDSCs correlated with high plasma viral loads and low CD4^+^ T cell counts, suggesting that depletion of BM MDSCs was associated with SIV/AIDS disease progression. Of note, in SHIV_SF162P4_-infected macaques, which naturally control viral replication within a few months of infection, the frequency of MDSCs in the bone marrow was unchanged. To investigate the mechanisms by which BM MDSCs were reduced during chronic SIV infection, we tested several hypotheses: depletion due to viral infection, alterations in MDSC trafficking, and/or poor MDSC replenishment. We found that the possible mobilization of MDSCs from BM to peripheral tissues and the slow self-replenishment of MDSCs in the BM, along with the viral infection-induced depletion, all contribute to the observed BM MDSC reduction. We first demonstrate MDSC SIV infection in vivo. Correlation between BM CD14^+^MDSC reduction and CD8^+^ T cell activation in tissues is consistent with decreased immune suppression by MDSCs. Thus, depletion of BM MDSCs may contribute to the pathologic immune activation during chronic SIV infection and by extension HIV infection.

## Author summary

Both cancer and infectious diseases including HIV/AIDS lead to the accumulation of myeloid-derived suppressor cells (MDSCs), which can effectively suppress anti-tumor and anti-viral T cell responses to dampen protective immunity. Using a macaque model, we found unexpectedly that the MDSCs in bone marrow (BM) decreased after chronic simian immunodeficiency virus (SIV) infection compared with healthy controls. This was in sharp contrast to the general increase of MDSCs observed in BM during cancer and other infectious/inflammatory diseases, and also contrary to the MDSC expansion in HIV/SIV-infected PBMCs. We further demonstrated that the loss of MDSCs in the bone marrow was associated with the progression to AIDS disease. Investigating the mechanisms by which the MDSCs were decreased in the SIV-infected bone marrow, we found that the possible mobilization of MDSCs from bone marrow to peripheral tissues and the slow self-replenishment of MDSCs in the bone marrow, along with the viral infection-induced depletion, all contribute to the observed bone marrow MDSC reduction. Indeed, this is the first demonstration to our knowledge of SIV infection of MDSCs in vivo. Because of the suppressive nature of the MDSCs, the CD8^+^ T cells might not be effective in killing the virally infected MDSCs. It is tempting to speculate that MDSCs may constitute latent reservoirs. Overall, our data showed that MDSCs act as a double-edged sword in HIV/SIV-infection, and the decrease of MDSCs in bone marrow after SIV infection could serve as an indicator of immune regulatory exhaustion and also contribute to the observed immune hyperactivation seen in HIV/AIDS.

## Introduction

Myeloid-derived Suppressor Cells (MDSCs) are immature cells of myeloid origin, frequently found in tumor microenvironments and in the blood of cancer patients [1–3]. Bone marrow (BM) is the reservoir for MDSCs. Under homeostatic conditions, there is a delicate balance between the immature MDSCs and matured myeloid cells in the BM. Normally, only matured myeloid cells are released from the BM to peripheral blood and tissues. MDSCs maintain a relatively low level in peripheral blood and tissues, and do not expand under normal physiological conditions. In healthy mice, Gr-1^+^CD11b^+^ MDSCs constitute 20–30% of the total cells in BM, 2–4% of peripheral blood cells, 2–4% of spleen cells, 2–5% of liver cells, and <1% of lymph node cells [2, 4, 5]. Cancer leads to MDSC expansion in tumors, and lymphoid tissues, reaching up to 50% of the total cells in lymph nodes, and bone marrow of tumor-bearing mice[6], and patients with pancreatic cancers [7]. MDSCs were implicated in the suppression of different immune cells including T cells and NK cells[8, 9]. Several suppressive mechanisms of MDSCs have been described including: (1) direct suppressive activities through the production of arginase1(ARG1), indoleamine 2,3-dioxygenase (IDO), reactive oxygen species(ROS), inducible nitric oxide synthase (iNOS), TGF-β, IL-10, and PD-L1 expression; (2) the expansion of regulatory T cells (Treg)[10–16]. In humans, MDSCs are a heterogeneous population without specific markers. Two main subtypes, monocytic, and polymorphonuclear (or granulocytic)-MDSCs, have been described in humans based on their phenotype and functions. Monocytic(M)-MDSCs were defined as HLA-DR^-/low^CD33^+^CD11b^+^CD14^+^ (hereafter referred to as CD14^+^ MDSC), while polymorphonuclear (PMN, also called granulocytic)-MDSCs were defined as Lin^-^HLA-DR^-/low^CD33^+^CD11b^+^CD15^+^(hereafter referred to as Lin^-^CD15^+^MDSCs) [8, 17–19]. Recently a new subset called early-stage MDSCs (eMDSCs) was added with phenotypical makers as Lin^-^(including CD3, CD14, CD15, CD19, CD56) HLA-DR^-/low^CD33^+^ [18]. In some of the studies, cancer leads to the expansion of both PMN and M-MDSCs, with PMN-MDSC as the predominant subset [19, 20]. Both subsets demonstrated a suppressive effect in vitro. However, M-MDSCs have been identified as the more suppressive subset in vivo, and the loss of PMN-MDSC has not altered tumor incidence [21]. MDSC expansion also occurs in non-cancer settings [12, 22–29]. For example, in late septic mice: Gr-1^+^CD11b^+^ MDSCs were increased dramatically with up to 40% of the total cells in spleen (vs. 2–4% in normal spleen), 90% of the total cells in BM (vs 20–30% in normal BM), and 3–4% of the total cells in lymph nodes (vs. <1% in normal lymph nodes)[30, 31].

MDSCs are significantly increased in peripheral blood of HIV/simian immunodeficiency virus (SIV)-infected individuals/macaques [12, 22, 23]. Similar to their roles in the tumor microenvironment, MDSCs in HIV/SIV-infections effectively suppress the function of T cells, and thus play an important role in dampening protective immunity [8, 12, 22, 23]. However, there are no details known regarding MDSC distribution and their roles in different tissues during HIV infection in humans. Because SIV-macaque models recapitulate the pathogenesis of HIV infections, they are considered one of the best animal models for AIDS research. In this study, we investigated MDSC tissue distribution, and possible MDSC roles during SIV infection using single suspension cells obtained from eight different anatomic compartments of SIVmac251-infected rhesus macaques. Surprisingly we found that MDSCs in the BM were paradoxically decreased in chronic SIV infection (dropping from 22% to 8%). Furthermore, the reduction of BM MDSC correlated with high plasma viral loads (VL), low CD4^+^ T cell preservation, and general high CD8^+^ T cell immune activation. We found that an increase in the trafficking and continuous mobilization of the MDSCs from bone marrow to peripheral blood and tissues might be one cause, along with poor replenishment of MDSCs in BM. A second cause is our discovery of viral infection of MDSCs that may further exacerbate the decrease of MDSCs. Taken together, our data suggest that the decrease in BM MDSC may contribute to immune deficiency and immune activation in macaques chronically infected with SIV.

## Results

### High frequencies of MDSCs in BM, liver, and PBMC, but not in lymph nodes (LN)

**Fig 1:**
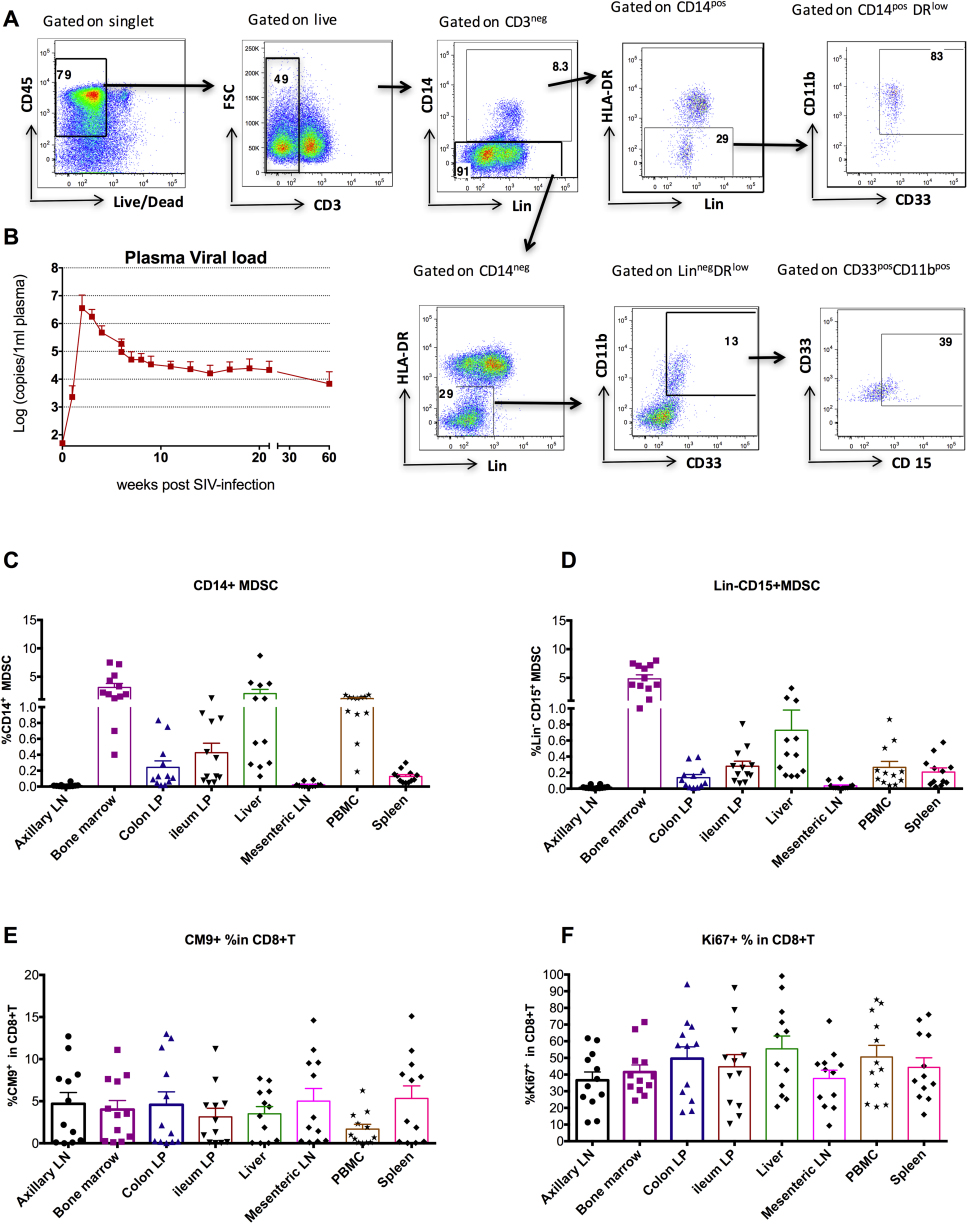
High frequencies of MDSCs were found in bone marrow, liver, and PBMC, but very few in the LNs of the chronically SIV-infected macaques. (A) The gating strategy of CD14^+^MDSCs and Lin^-^ CD15^+^MDSCs from the SIV-infected bone marrow. The same gating strategy was used for other compartments. (B) The kinetics of plasma VLs in the 12 SIV_mac251_-infected macaques showed persistent viral infection (mean±SEM). Single suspension cells from eight compartments of 12 SIVmac251 chronically (14 months-) infected Indian rhesus macaques were used to measure the frequencies of CD14^+^ MDSCs (C), Lin^-^ CD15^+^MDSCs (D), gag-specific CM9 Dextramer^+^ (E), and Ki67^+^ (F) in CD8^+^ T cells. In C-D, P<0.05 for bone marrow vs. all the other compartments except liver and PBMC; P<0.05 for liver vs. axillary LN, mesenteric LN, and colon LP; P<0.05 for PBMC vs. axillary LN, mesenteric LN, and colon LP in both types of MDSCs after Dunn’s multiple comparison tests. In E, P<0.05 for PBMC vs. axillary LN, mesenteric LN, spleen and bone marrow after Dunn’s multiple comparison tests.

Because information on tissue distribution of the MDSCs in normal and HIV-1 infected humans is limited, we investigated the distribution of MDSCs in eight different tissue compartments of SIVmac251-infected Indian rhesus macaques. Single cell suspensions from tissues of 12 SIVmac251 chronically infected macaques (14 months post-infection) were obtained and subjected to flow cytometric analysis of MDSCs [23]. We examined the frequencies of two MDSC subsets, which were both HLA-DR^-/low^CD33^+^CD11b^+^ and either Lin^-/low^ CD15^+^(Lin^-^CD15^+^MDSC) or CD14^+^ (CD14^+^ MDSC) (Fig 1A) [19, 23, 32–34]. These animals showed a typical dynamic of the plasma VL after pathogenic SIVmac251 infection with peak VL at Week 2 followed by persistent set-point VL from Week 6 to the end study at week 60 (Fig 1B). Among the eight compartments from the chronically infected macaques, interestingly, high frequencies of both types of MDSCs were found in BM (4.8% and 3.1% for Lin^-^CD15^+^ and CD14^+^ MDSCs, respectively, P<0.05 for BM vs. all the other compartments except liver and PBMC in both types of MDSCs after Dunn’s multiple comparison tests), liver (0.8% and 2.0% for Lin^-^CD15^+^ and CD14^+^ MDSCs, P<0.05 for liver vs. axillary LN, mesenteric LN, and colon lamina propria (LP) in both types of MDSCs after Dunn’s multiple comparison tests), and PBMC (0.3% and 1.1% for Lin^-^ CD15^+^ and CD14^+^ MDSCs, P<0.05 for liver vs. axillary LN, mesenteric LN, and colon LP in both types of MDSCs after Dunn’s multiple comparison tests) with intermediate levels of MDSCs in spleen and gut; but very few of either subset in the LNs (Fig 1C and 1D). For both subsets of MDSCs, when we analyzed the associations among different tissues, we could not find any correlations between any pairs of compartments (S2 and S3 Tables), suggesting compartmentalization. For example, the animal with the highest CD14^+^MDSCs in the BM (8%) only had 0.2% of CD14^+^MDSCs in the PBMC.

In addition to MDSCs, we also measured viral-specific CD8^+^ T cell responses by gag CM9 peptide-Mamu A*01 Dextramer as well as the total proliferating CD8^+^ T cells by Ki67 in these animals, because both can be affected by MDSCs. Because all the animals were Mamu A*01^+^, SIVgag-CM9 Dextramer^+^ cell frequencies were measured to represent viral-specific CD8^+^ T cell responses[35]. As shown in Fig 1E, SIVgag-CM9 Dextramer^+^ cell frequencies in PBMC was 1.7%, much lower than in the remaining tissues, which ranged from 3% to 5% of total CD8^+^ T cells (P<0.05 for PBMC vs. axillary LN, mesenteric LN, spleen and BM after Dunn’s multiple comparison tests). However, the frequencies of CM9 Dextramer^+^ in total CD8^+^ T cells from different tissues were highly correlated with each other (S4 Table), suggesting the equilibration of antigen-specific T cell responses among different tissue compartments. The proportion of total CD8^+^ T cells that expressed Ki67 ranged from 35 to 54%, without significant differences among the compartments (Fig 1F). We found that the frequencies of Ki67^+^ within the total CD8^+^ T cells from different tissues were also associated with each other (S5 Table). Overall, the tissue distribution patterns of viral-specific CM9 Dextramer^+^ and total Ki67^+^ CD8^+^ T cells were different from those of MDSCs in the same SIV-infected macaques. It is of note that the MDSCs did not accumulate in the lymphoid tissues of the SIV-infected macaques during the chronic stage, which marked a sharp contrast to cancer and some infectious diseases[36].

### MDSCs in bone marrow were decreased after SIV infection, and correlated with markers of SIV disease progression

**Fig 2:**
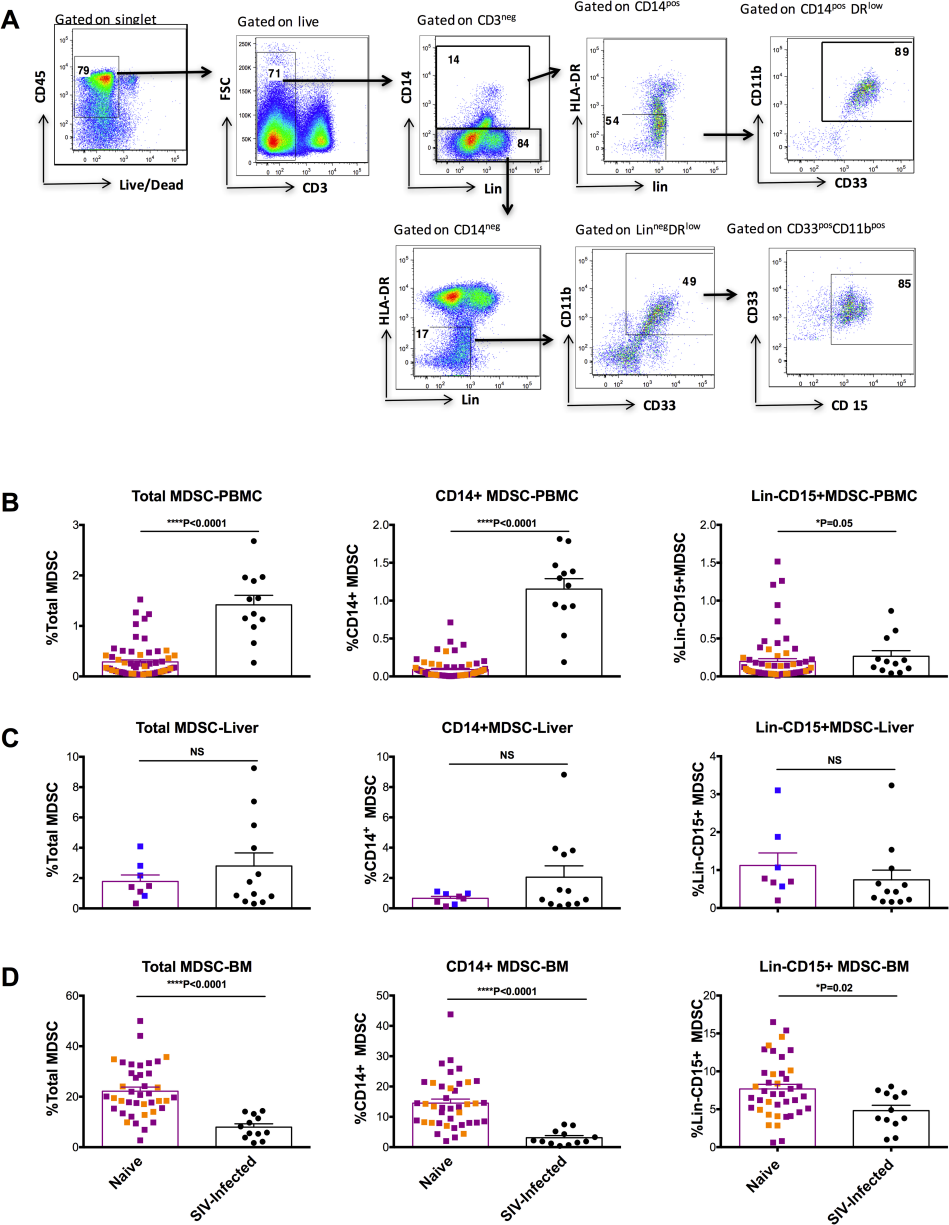
CD14^+^ and Lin^-^CD15^+^MDSCs were decreased in BM and increased in peripheral blood during chronic SIV infection. (A) The gating strategy of CD14^+^MDSCs and Lin^-^CD15^+^ MDSCs from the naïve bone marrow. (B-D) The MDSC frequencies of the naïve macaques in the PBMCs (68 naïve animals, the purple and orange squares denote the PBMC samples from two different cohorts of naïve animals.), liver (8 animals; purple and blue squares represent livers from 2 different cohorts of naïve animals) and bone marrow (40 naïve animals, the purple and orange squares denote the bone marrow samples from two different cohorts of naïve animals.) were measured and compared with those of the SIV-infected macaques (n = 12 for each compartments). Mann-Whitney was used for the comparisons.

Due to the tissue distribution shown in Fig 1, we then focused on the MDSCs in BM, liver and PBMC. One important question was whether the frequencies of the MDSC subsets were altered during chronic SIV infections. To make the comparisons, we measured the frequencies of MDSCs among PBMCs (from two cohorts, n = 40 and 28), liver (from two cohorts, n = 4 and 4) and BM (from two cohorts, n = 28 and 12) from naïve macaques. Using a similar gating strategy (Fig 2A), we found that while naïve animals had a low frequency of MDSCs in PBMCs (0.09±0.02% and 0.20±0.04% for CD14^+^ and Lin^-^CD15^+^MDSC), PBMC MDSC frequencies were elevated after SIV infection (1.15±0.14% and 0.27±0.08% for CD14^+^ and Lin^-^CD15^+^MDSC in PBMCs) (Fig 2B). This is consistent with the results from previous studies showing an expansion of MDSCs in the PBMCs after HIV-1/SIV infection[12, 22, 23]. Though both subsets of MDSCs were more prevalent, we observed a more dramatic increase of the CD14^+^ MDSC subset than the Lin^-^CD15^+^MDSC subset: 13-fold increase of CD14^+^ MDSC compared with almost no change of Lin^-^CD15^+^MDSC after SIV chronic infections. Due to the small number of naïve liver samples that were available, we could not reach any conclusions as to effect of SIV infection on liver MDSCs (Fig 2C). Surprisingly, we found that the total number of MDSCs (defined as the sum of CD14^+^ MDSC and Lin^-^CD15^+^MDSC) in BM was paradoxically decreased 14 months after SIVmac251 infection (Fig 2D). Specifically, there was roughly a 4.7-fold reduction in the CD14^+^ MDSC subset (from 14.5±1.3% in naïve animals vs. 3.1±0.07% in SIV-infected ones, P<0.0001), and a 1.6-fold reduction in the Lin^-^CD15^+^MDSC subset (7.7±0.6 vs. 4.8±0.7% respectively, P = 0.005) (Fig 2D). This pattern of the MDSC distribution in the SIV-infected animals was markedly different from that in tumor-bearing mice, where BM MDSCs are elevated [4, 5]. Furthermore, we found two subpopulations of CD14^+^ MDSCs in the BM: CD14^high^ and CD14^intermediate^ MDSCs (S1 Fig). Most CD14^intermediate^ population were HLA-DR^-/low^ cells in SIV-infected and naïve BM. This HLA-DR^-/low^ CD14^intermediate^ population was prominent in the naive animals, but lower in the SIV-infected animals (S1 Fig). Compared to naïve BM, the CD14^+^ population was also decreased in the SIV-infected BM (S2 Fig).

Because MDSCs, especially Lin^-^CD15^+^ subset, are sensitive to cryopreservation [37], and we used frozen cells throughout the whole study, there is a possibility that SIV infection might differentially affect the loss of SIV-infected MDSCs compared to naïve cells. To test this, we compared the frequencies of MDSC subsets in paired specimens of fresh vs frozen BM and PBMC obtained from the SIV-infected (BM: n = 6; PBMC: n = 7) and naïve animals (BM: n = 12; PBMC: n = 5). The PBMC and BM samples from the SIV-infected and naïve animals were isolated, and divided into two aliquots, one was immediately stained and analyzed by flow-cytometry, the other was cryopreserved in liquid N_2_ and thawed for testing 4–5 months later. After cryopreservation and thawing, 70–80% of CD14^+^MDSCs were preserved in the naïve and SIV-infected BM samples; 65% of CD14^+^MDSCs were also preserved in the SIV-infected PBMC samples (S3 Fig). Consistent with the previous study [37], only 20% of Lin^-^CD15^+^ MDSCs were preserved in the naïve and SIV-infected BM samples (S3 Fig), suggesting Lin^-^CD15^+^MDSC subset was more sensitive to cryopreservation. Importantly, we did not observe any significant difference of MDSC subset loss for the BM samples between the SIV-infected and naïve animals. Thus, SIV infection does not differentially affect the loss of MDSC subsets from BM compared to naïve ones.

**Fig 3:**
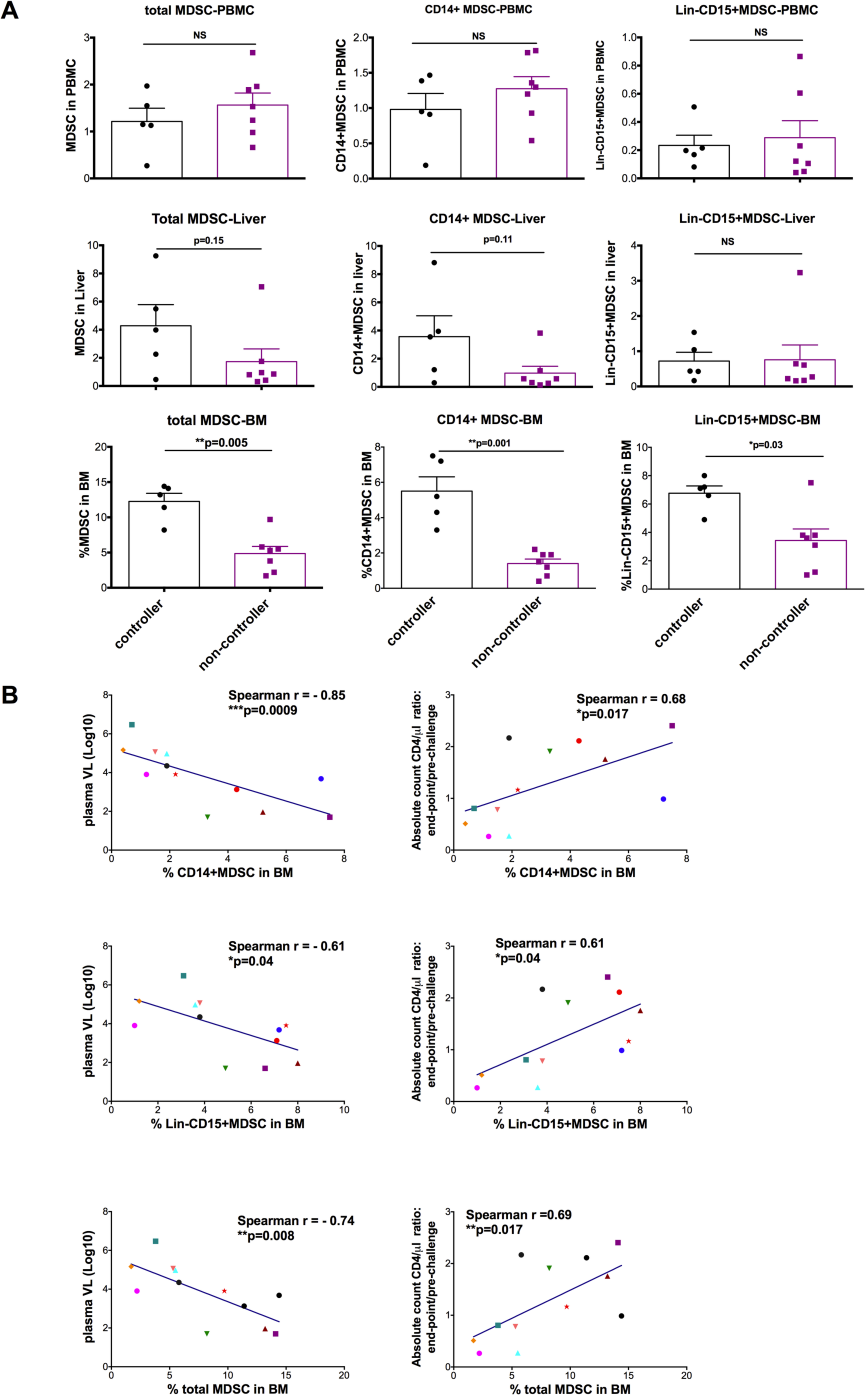
After chronic SIV-infection, non-controller (log VL>3.7) had lower MDSC in the bone marrow than those of the controllers (log VL<3.7). (A) Among the 12 SIV-infected macaques, 5 of them were controllers with plasma viral loads below 5,000 copies/ml, while the remainder of them (n = 7) were non-controllers with viral loads greater than 5,000 copies/ml. MDSC (both CD14^+^ MDSC and Lin^-^CD15^+^ MDSC subsets) in the bone marrow of the controllers maintained relatively higher levels compared with those of the non-controllers. Mann-Whitney was used for the comparisons. (B) The MDSCs in the bone marrow inversely correlated with the plasma VLs, and positively correlated with absolute CD4 count ratio (Necropsy CD4^**+**^ T cell count/pre-challenge CD4^+^ T cell count). Spearman analysis was used for correlations. Total MDSC was the sum of CD14^+^MDSC and Lin^-^CD15^+^ MDSC subsets.

We then asked whether SIV VLs correlated with the reduction of BM MDSCs. Among the 12 SIV-infected macaques, five of them controlled SIV replication, with plasma VLs below 5,000 copies/ml. The remaining 7 animals did not control SIV replication, with VLs >5,000 copies/ml. We found that the controllers maintained high BM MDSC frequencies, whereas the non-controllers had reduced BM MDSC frequencies (Fig 3A). This change was not found in the PBMC or the liver, although the liver showed the same trend (Fig 3A). Indeed, the frequencies of both subsets of MDSCs in BM inversely correlated with VL (Fig 3B). In the BM of chronically SIV-infected macaques, the frequencies of CD14^intermediate^ MDSCs inversely correlated with plasma viral loads better than that of CD14^high^ MDSCs (S4 Fig). We also found that both subsets of MDSCs in the BM positively correlated with absolute blood CD4 count ratio (necropsy end-point CD4^+^ T cell count/pre-challenge CD4^+^ T cell count ratio) (Fig 3B). The association between BM MDSC depletion and high VLs and low CD4^+^ T cell counts suggests that MDSC depletion may contribute to disease progression, or at least serve as marker of disease progression.

### SHIV_SF162P4_-infected macaques, which had undetectable plasma VL 11 months post-infection, maintained their normal frequency of MDSCs in bone marrow

**Fig 4:**
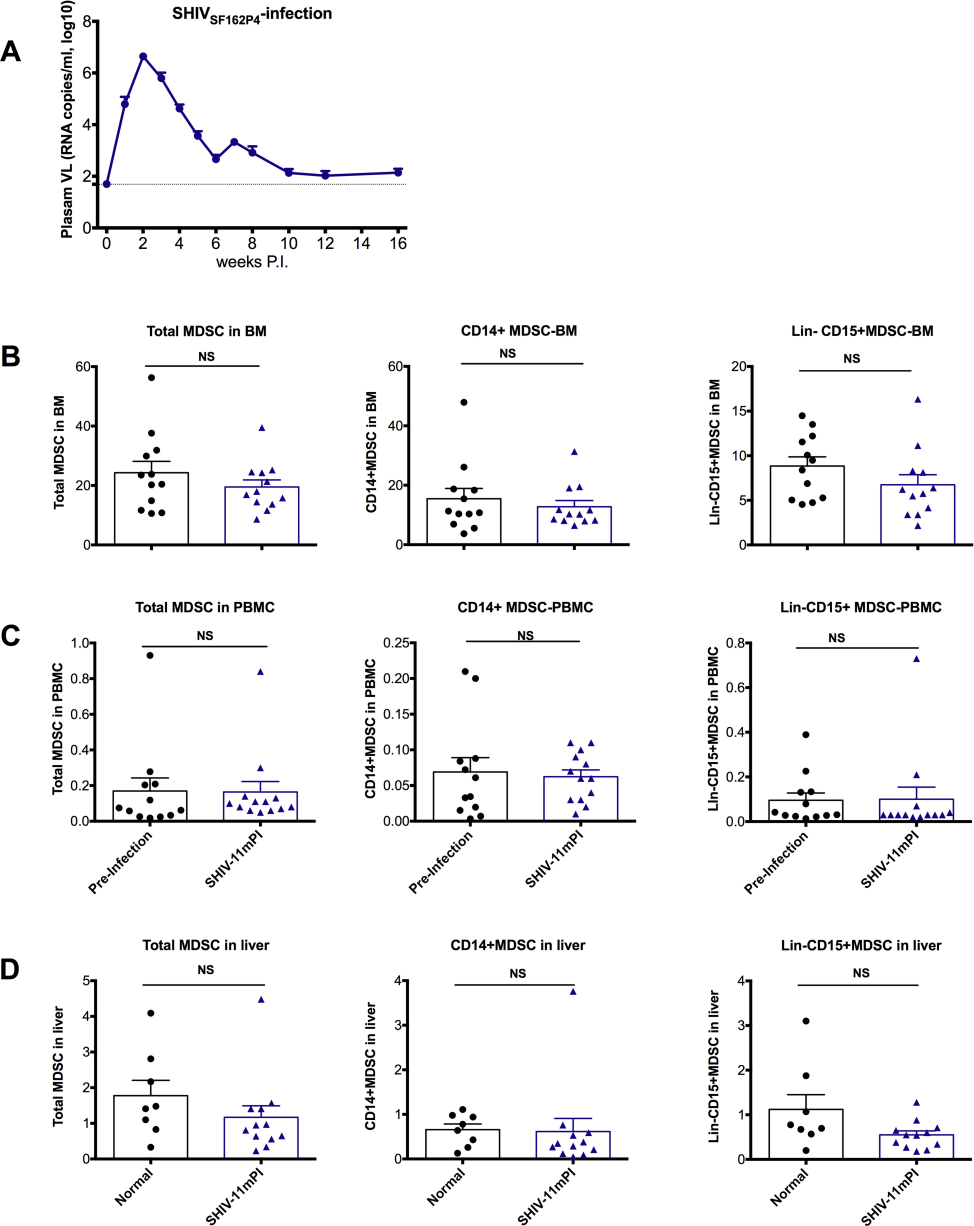
The MDSCs in the bone marrow of macaques after SHIV-infection showed that persistent viral exposure was needed for the reduction of the MDSCs in the bone marrow. (A) Plasma VLs after SHIVSF162P4-infected macaques (n = 12) (mean±SEM). Total MDSCs CD14^+^ MDSCs, and Lin^-^CD15^+^ MDSCs in bone marrow (B), and PBMC (C) of the macaques before infection, and 11 months post-SHIV infection were compared. Pre-infection samples of PBMC and BM were the matched controls for the 11-month post-infection samples. Wilcoxon tests were used for comparisons. (D) The MDSCs in the liver of the 11 months post-SHIV infections were compared with naïve and normal controls, Man-Whitney were used for comparisons.

The data suggested that exposure to pathogenic SIV infection decreased MDSC frequencies in the BM. We then asked if similar changes occurred in Indian rhesus macaques infected with SHIV_SF162P4_. All the SHIV_SF162P4_-infected animals demonstrated high peak VLs during the first 6 weeks of infection, followed by viral control 3 to 4 months post-infection (Fig 4A). At 11 months post-infection, when VL was near or below the detection limit, there was no change in BM MDSC frequency (Fig 4B). In those samples, there was no change in either the Lin^-^CD15^+^MDSC or CD14^+^ MDSC (Fig 4B). The same was also true for MDSCs in the 11-month post SHIV-exposed PBMC and liver (Fig 4C and 4D). Thus, infection with SHIV without persistent viremia did not affect MDSC frequency in BM, but infection with pathogenic SIV depleted BM MDSC.

### Mechanisms of MDSC reduction in the bone marrow of macaques chronically infected with SIV

We next investigated what caused the reduction of MDSCs especially in the CD14^+^ subset in the BM of the SIV-infected macaques. We proposed three hypotheses: depletion due to viral infection, increase in MDSC mobilization/trafficking out of BM, and poor MDSC replenishment from hematopoietic stem cells.

#### MDSC infection by SIV in vivo

**Fig 5:**
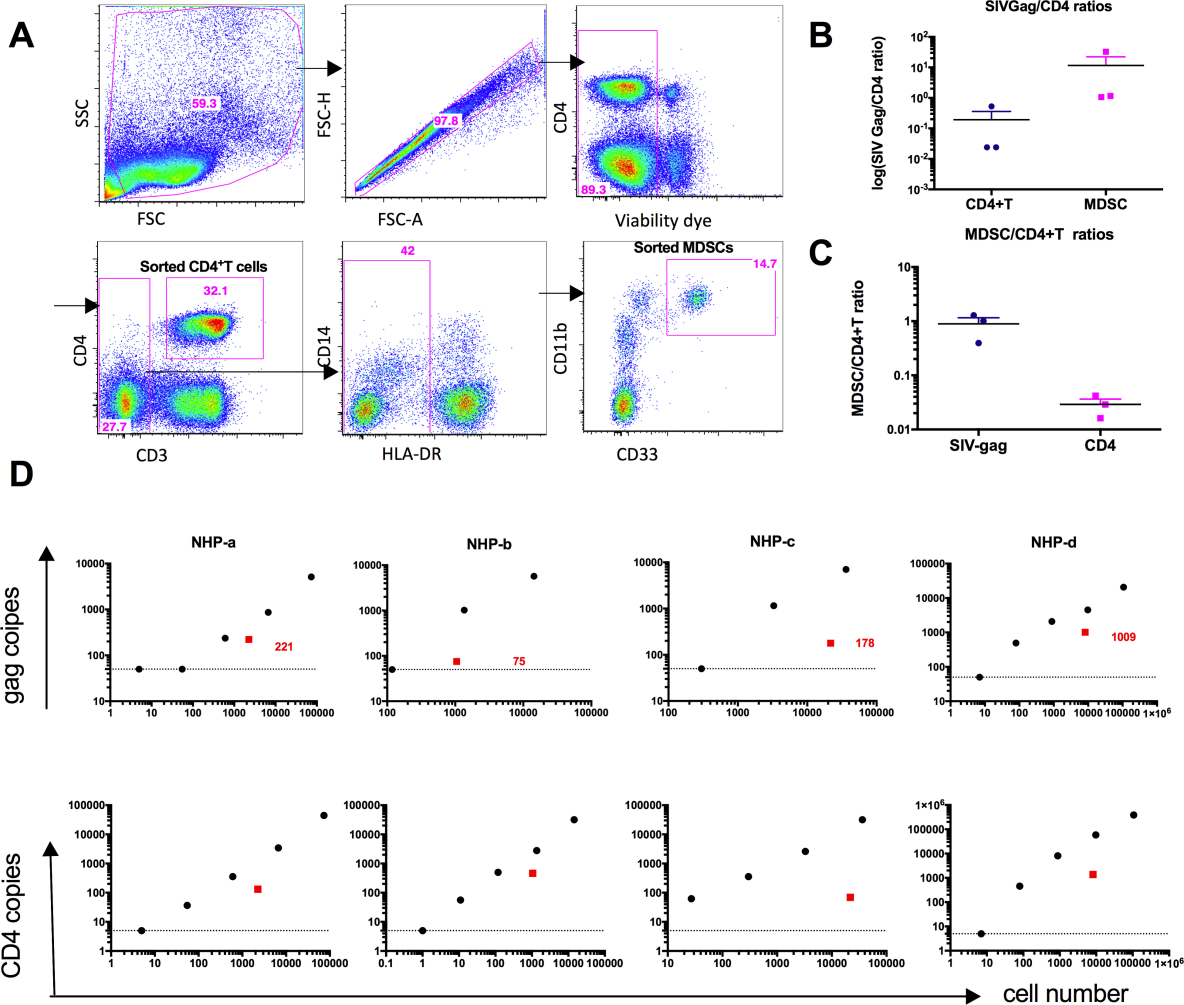
qPCR analysis of SIV-gag showed that the MDSCs were infected by SIVmac251 in vivo. (A) Sorting strategies for MDSCs and CD4^+^T cells from the PBMCs of SIV-infected macaques. (B) The ratio of mRNA of SIVgag vs mRNA of CD4 in CD4^+^T cells and MDSCs showed that MDSCs had a higher SIVgag/CD4 ratio. (C) The ratio of SIVgag expression level in MDSCs vs SIV gag expression level in CD4^+^T cells showed that MDSCs and CD4^+^T cells had a comparable level of SIVgag expression. (D) In another independent experiment, MDSCs and CD4^+^ T cells from the PBMCs of the SIV-infected animals were sorted as in A. For the sorted CD4^+^ T cells, the samples were serially diluted (10–fold) before the RNA isolation. After RNA isolation and cDNA synthesis, each of the CD4^+^ T and MDSC samples was subjected to qPCR analysis for SIVgag, CD3, CD4, and GAPDH using the same amounts of cDNA. The detection limit for Gag and CD4 is 50 copies/well (shown by the dashed horizontal lines). If the gag copy number was lower than 50, 50 was assigned to the tested sample. Black dots in D are CD4^+^ T cells as positive control titration curve, while red squares are the MDSCs plotted on the same scale, and red numbers are the copy numbers of SIVgag in MDSCs. The number of cell equivalents used in each assay was shown in the horizontal axis.

The SHIV infection data suggest that only pathogenic viruses can decrease MDSC frequency. Thus, the MDSC decrease may be due in part to direct infection with SIV and virus-mediated cell death. In fact, HIV-1 infects MDSCs *in vitro* [22]. We asked whether MDSCs were infected by SIV *in vivo*, which had not been described previousl*y*. Using FACS, we purified MDSCs from the PBMCs or spleens of the chronically SIV-infected macaques (Fig 5A, purity>95%). The isolated RNAs from purified MDSCs were then subjected to qPCR to detect the expression levels of SIV gag, CD3 and CD4, while purified CD4^+^ T cells were used as positive controls. We found that all the MDSC samples were negative for CD3 mRNA, suggesting no T cell contamination for the MDSC subsets. Most of the MDSC samples (4 of 6) were positive for SIV gag and CD4, indicating infection of the MDSCs by SIVmac251. Importantly, the ratio of SIVgag to CD4 was much higher in the MDSC population than the CD4^+^ T cell population, further confirming that the virus detected could not be due to contaminating CD4^+^ T cells (Fig 5B, S5 Fig). Concordant with that, we also found that the SIVgag expression level is comparable between CD4^+^ T cells and MDSCs (ratio around 1), whereas the CD4 expression level was much lower in MDSCs than in the CD4^+^ T cell subset (Fig 5C, S6 Fig), further supporting the conclusion that MDSC-associated viral RNA could not be due to CD4^+^ T cell contamination. Since the SIVgag assay cannot distinguish the genomic RNA in virions or infected cells from the SIVgag mRNA, we performed real-time RT-PCR assays to detect the spliced viral mRNAs that are produced only in infected cells. Spliced viral mRNAs can thus act as a marker of active virus replication. While all the T cell samples were positive for spliced viral mRNA, out of the four MDSC samples that were positive for SIVgag, we found that 2 of them were also positive for spliced viral mRNA (S7 Fig). Given the importance of this finding, we tested additional animals in an independent experiment to confirm the conclusion of productive infection of MDSCs. We performed another MDSC sorting using six SIVmac251-infected BM samples to detect the spliced SIV mRNA. The sorted CD4^+^T cells were 10-fold serially diluted to generate a titration curve so that the detection limit of spliced SIV /CD3 mRNA was from 90 to 900000 CD4^+^T cells. Four sorted MDSC samples were negative for CD3 mRNA, and three out of these four were positive for spliced SIV mRNA (S7 Fig). Overall, these two independent experiments (S7 Fig) demonstrated that the spliced SIV mRNA were present in the MDSC (5 out of 8 CD3 negative) samples, indicating productive infection rather than BM-phagocytosis of infected cells.

To further confirm that SIV infects MDSC in-vivo, we sorted MDSCs and CD4^+^ T cells from the blood of 6 additional SIV-infected animals. We isolated RNA from MDSC and CD4^+^ T cell samples and performed 10-fold serial dilutions of CD4^+^ T cells (up to 5 dilutions) to generate titration curves, which were linear on a log-log scale (black dots in Fig 5D). After RNA isolation and cDNA synthesis, each of the CD4^+^ T and MDSC samples was subjected to qPCR analysis for detection of SIVgag, CD3, CD4, and GAPDH using the same amounts of cDNAs (red symbols superimposed on titration curves represent MDSCs from the same animal on the same scale). Out of these 6 animals, we failed to isolate RNA from one sample due to a low number of MDSC sorted. Out of the five samples from which we have successfully isolated RNA, we could not detect SIVgag from one sorted MDSC sample (the detection limitation for Gag is 50 copies/well). Among the four MDSC samples scoring positive for SIVgag, we detected CD3 mRNA from two of them (NHP-a and -b) (Fig 5D), indicating possible contamination or phagocytosis of the T cells as suggested by another study[38]. However, SIV gag was detected in the two remaining SIVgag-positive samples (NHP-c and–d), that were negative for CD3 mRNA, indicating infection of MDSC. The sensitivity to detect T cell contamination in these two animals was 27 and 80 CD4^+^ T cells (S7 Fig). Note that the housekeeping gene GAPDH was expressed at the same level per cell in the MDSCs (red) as in the CD4^+^ T cells (black dots) (S7 Fig).

**Fig 6:**
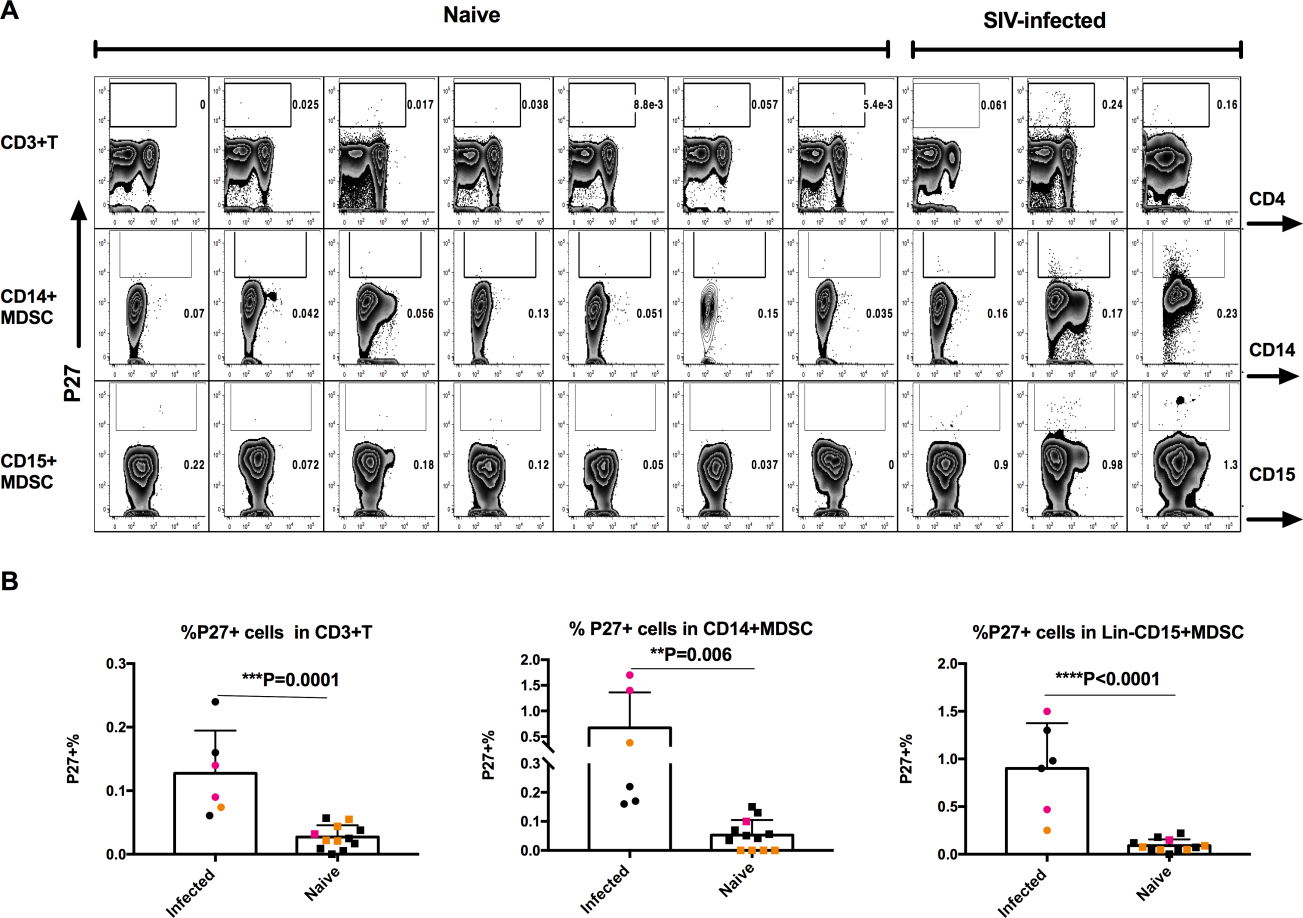
Flow cytometric detection of gag-P27^+^ MDSCs and CD4^+^T cells in the bone marrow of SIV-infected or naive animals. (A) One representative P27 staining from one experiment with 7 naïve and 3 SIV-infected animals (B) Summary of three independent experiments with total 6 SIV-infected, and 12 naïve animals. The SIV-infected and naïve animals from the same experiment were shown in same color. Each data point represents one individual animal. The Man-Whitney tests were used for comparisons.

We also assessed SIVgag p27 expression in BM cells of SIVmac251 chronically infected BM by Immunocytochemistry. In all 6 SIV^+^ animals a variable number of Lin^-^CD15^+^ and CD14^+^ MDSCs were positive for SIV P27. Thus, both subsets of MDSC are infected by SIVmac251 in-vivo (Fig 6). Results from one representative experiment are shown (Fig 6A). The summary of three independent experiments demonstrated that the P27^+^% in both subsets of MDSCs from the SIV-infected bone marrow was significantly higher than in naïve animals (Fig 6B, S8 Fig). Note that the MDSCs from SIV-infected animals not only had substantially more events in the positive gate, but also had brighter staining indicating real infection. Thus, SIV infects both subsets of MDSCs *in vivo* and this could directly contribute to their depletion from the BM of infected animals.

#### MDSC trafficking from BM to blood

We then explored the MDSC mobilization/trafficking hypothesis, in which MDSCs moved from BM to blood and peripheral tissues with an accelerated rate after SIV-infection compared with those of the uninfected controls.

**Fig 7:**
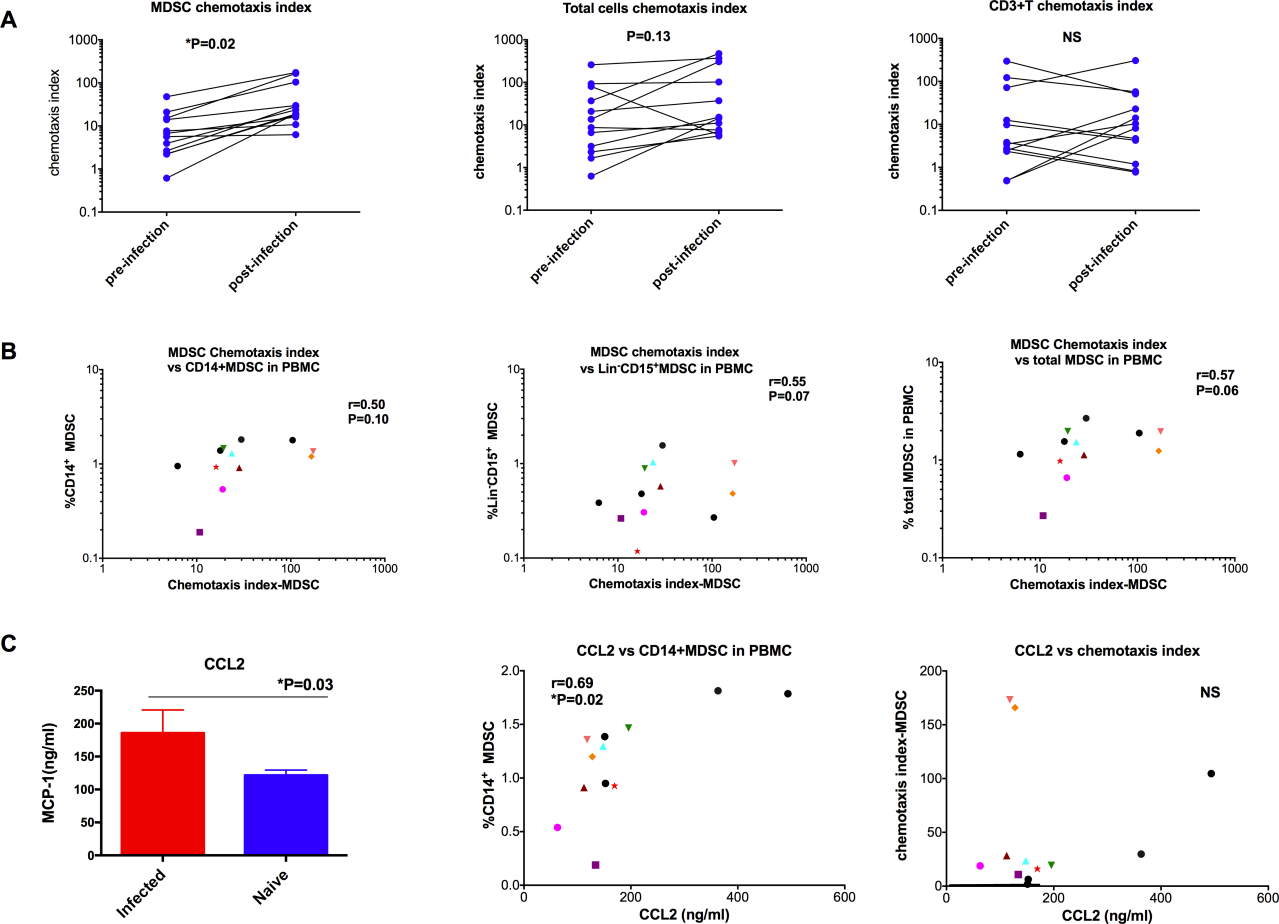
Preferential trafficking of MDSCs from BM to peripheral blood during chronic SIV infection measured by in vitro chemotaxis assays. (A) Chemotaxis index of MDSCs, T cells and total cells were compared before and after SIV infections. Wilcoxon tests were used for comparisons. (B) Correlation trends were observed between MDSC chemotaxis index and CD14^+^ or Lin^-^ CD15^+^ or total MDSCs in the PBMCs after chronic SIV infection. Spearman analysis was used for correlations. (C) Plasma CCL2 level was higher in the SIV-infected macaques than in naïve ones, and correlated with CD14^+^ MDSC in the PBMCs, but did not correlate with the MDSC chemotaxis index. Man-Whitney tests were used for comparisons, and Spearman analysis was used for correlations.

To test this hypothesis, we performed *in vitro* chemotaxis assays to measure the movement of BM single-cell suspensions towards the plasma of pre- and post-infected animals. We observed that the MDSC chemotaxis index toward the plasma of the post-infected samples was significantly higher than those of the pre-infected plasma (Fig 7A). However, the chemotaxis indexes of total cells and of T cells did not change after SIV-infection (Fig 7A). These data suggested a preferential trafficking of MDSCs, but not T cells or total cells, from BM to blood after SIV-infection. We then investigated whether the MDSC chemotaxis index was associated with the frequencies of MDSC in the SIV-infected PBMC. We observed trends of positive correlations between the MDSC chemotaxis index from the SIV-infected plasma and the frequencies of CD14^+^, CD15^+^Lin^-^, and total MDSCs in SIV-infected PBMCs, suggesting the important role of MDSC trafficking from bone marrow to blood during SIV infection (Fig 7B).

We then sought to characterize the chemokine(s) responsible for the trafficking of MDSCs. During cancer and HIV infection, the balance between MDSCs in BM and peripheral tissues is interrupted due to the massive cytokine/chemokine storm that characterizes these conditions. CCL2 (also known as MCP-1) is a key chemokine involved in MDSC trafficking. In tumor-bearing mice, the CCR2-receptor is highly expressed by most MDSC subsets, and CCL2 alone is sufficient to induce migration of MDSCs [39]. It has been shown that CCL2 is up-regulated upon HIV infection [40]. Based on this, we proposed that changes in CCL2 levels might be responsible for the trafficking of the MDSCs from BM to the periphery in the SIV-infected macaques. Indeed, we found that the expression level of CCL2 in the plasma of SIV chronically infected macaques was significantly greater than that of the naïve control samples (Fig 7C). Furthermore, it was also positively correlated with the CD14^+^ MDSC subset (Fig 7C), but not the Lin^-^CD15^+^MDSC (Spearman P = 0.9) in the PBMCs, suggesting that CCL2 might be responsible for the accumulation of the CD14^+^ MDSC in the peripheral blood. However, we did not find a correlation between CCL2 and the MDSC chemotaxis index measured in *in vitro* chemotaxis assays, suggesting that other chemokines, besides CCL2, in the SIV-infected plasma, play roles in determining the migration of MDSCs from BM to blood (Fig 7C).

#### MDSC replenishment from hematopoietic stem cells

**Fig 8:**
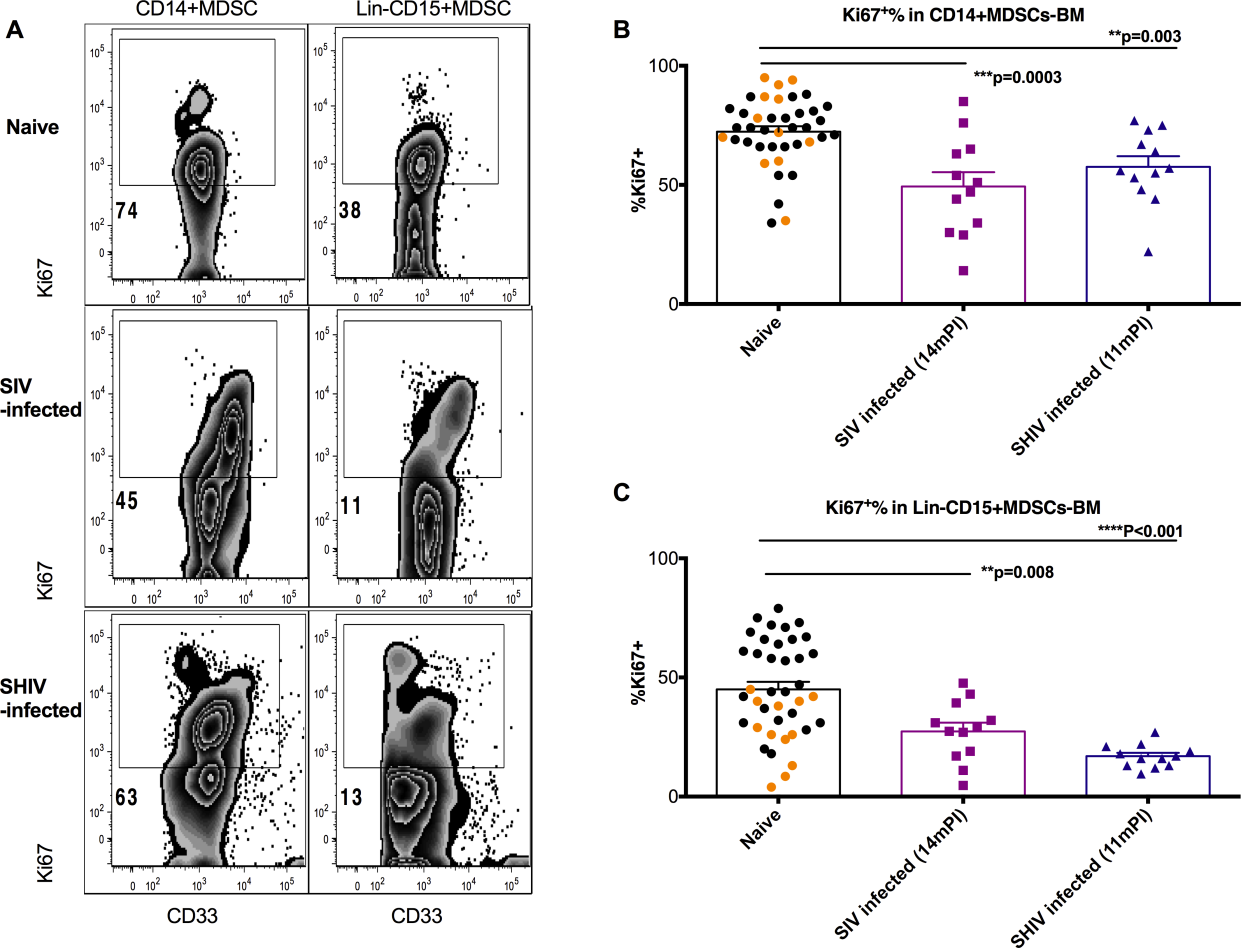
MDSC proliferation was decreased in the bone marrow of SIV-infected macaques. (A) Representative Ki67^+^% of the gated CD14^+^ and Lin^-^ CD15^+^MDSCs in the bone marrow from naïve, SIV- or SHIV-infected macaques. (B, C) Ki67^+^% of the gated CD14^+^ and Lin^-^ CD15^+^MDSCs in the bone marrow was lower in the SIV-infected macaques than in the naïve, or SHIV-infected animals. Man-Whitney were used for comparisons between the naïve and SIV- or SHIV-infected macaques. The black and orange dots denote the bone marrow samples from two different cohorts of naïve animals.

Another important factor that could affect the frequency of the MDSCs in the bone marrow is rate of MDSC replenishment. Due to the infection of the BM accessory cells, impaired stromal function and alteration of the hematopoietic growth factor network was frequently observed in the BM of patients with HIV-1 infection[41–43]. These events directly lead to disruption of normal hematopoiesis in the BM[44, 45]. Our hypothesis was that chronic SIVmac251 infection might impair the proliferation of MDSCs in the BM. We measured the cell proliferation marker Ki67 to assess the proliferative replenishment rate of MDSCs in the BM. Indeed, proliferation of both CD14^+^ and Lin^-^CD15^+^MDSCs in BM was lower in SIV-infected macaques than in naïve controls (Fig 8A–8C). In the SHIV-infected macaques, which spontaneously controlled their viral replication several months after the infection, we found that the proliferation of the CD14^+^ and Lin^-^CD15^+^ MDSCs in the bone marrow 11 months post-infection were also significantly lower than those of the uninfected controls (Fig 8A–8C). Even through the proliferation of both subsets of MDSCs were decreased in SHIV chronically infected BM, the frequency of both subsets were maintained at pre-infection levels (Fig 4B). Interestingly, in both SIV-and SHIV-infected animals, we observed a bimodal Ki67-positive population Ki67^high^ subsets in CD14^+^ and Lin^-^CD15^+^MDSCs, which were almost absent from those of the uninfected controls (Fig 8A).

Thus, the slow self-replenishment of BM MDSCs probably exacerbated the decrease of MDSCs when they were killed by virus, or migrated out of the BM.

### MDSC reduction in bone marrow was associated with increased T cell immune activation

**Fig 9:**
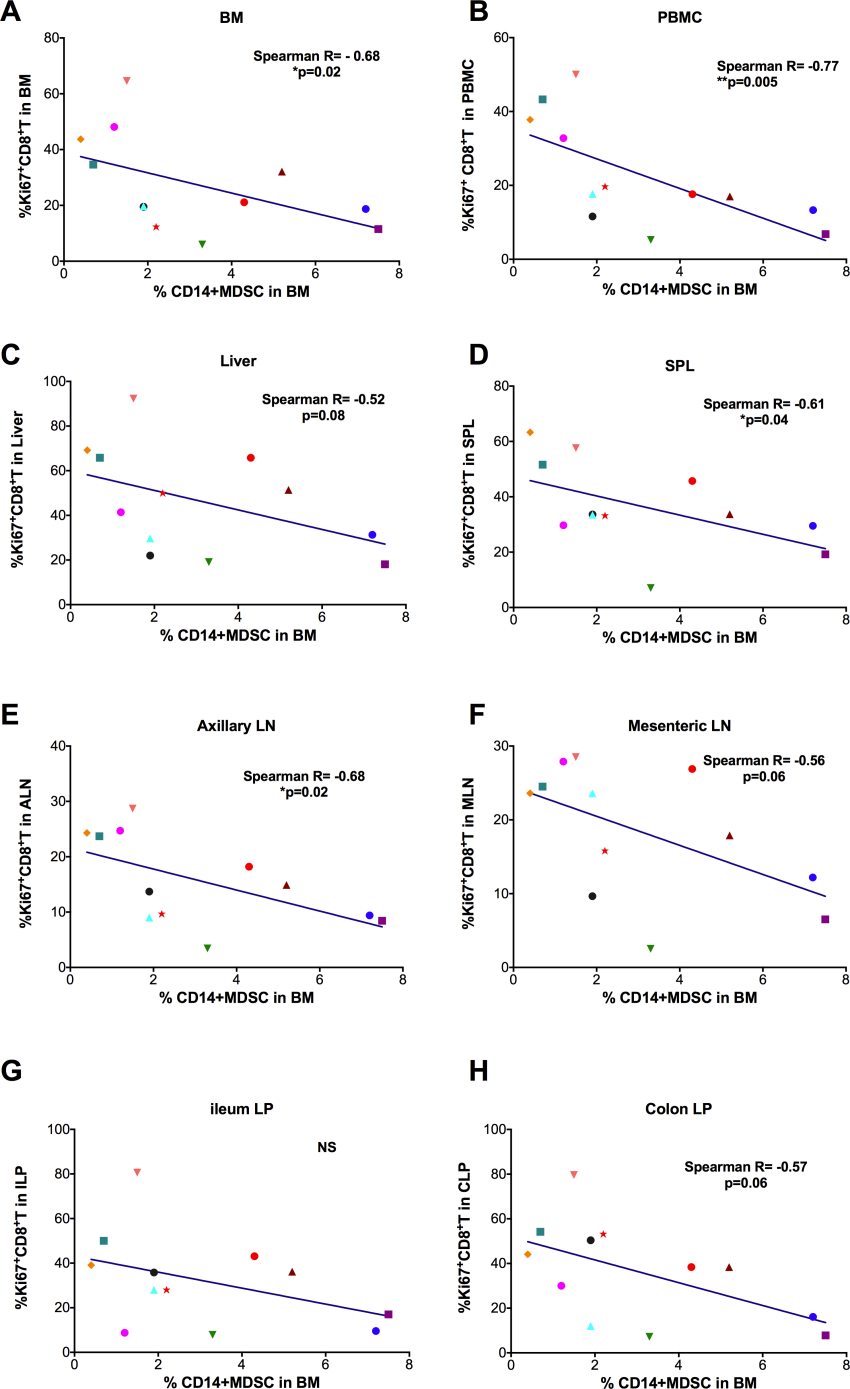
Decreased CD14^+^ MDSCs in bone marrow were associated with increased immune activation. The frequency of CD14^+^ MDSCs was inversely correlated with the Ki67^+^ CD8^+^ T cell frequencies in the SIV-infected bone marrow (A), PBMC (B), spleen (D), Axillary LN (E) and Mesenteric LN (F), but not liver (C), ileum LP (G) or colon LP (H). (although MLN and colon LP showed strong trends (p = 0.06). Each data point represents one animal. Spearman analysis was used for correlations.

We then sought to clarify the consequences of the MDSC reduction in the BM. Because the main known function of MDSCs is to limit excessive T cell proliferation, and thus control tissue damage, we proposed that BM MDSC reduction would be associated with T cell activation, especially among CD8^+^ T cells. We measured the frequencies of Ki67^+^ CD8^+^ T cells in the tissues of the SIV-infected macaques, and then performed correlation analyses between frequency of BM CD14^+^ / Lin^-^CD15^+^MDSCs and the frequencies of Ki67^+^ CD8^+^ T cells in these compartments. We found that the frequency of BM CD14^+^ MDSCs inversely correlated with the frequency of Ki67^+^CD8^+^ T cells in BM, PBMC, spleen, ALN, but not MLN, liver, ileum LP and colonic LP (although MLN and colonic LP showed nearly significant trends) (Fig 9A–9H); whereas Lin^-^CD15^+^ MDSC did not correlate with the frequency of Ki67^+^CD8^+^ T cells in any of the compartments except PBMC (S9 Fig). Overall, the association of BM CD14^+^ MDSC frequency with CD8^+^ T cell immune activation, plasma vRNA levels, and CD4^+^ T cell loss suggests that the reduction in BM CD14^+^ MDSC contributes to the pathogenesis of the HIV/SIV infections.

## Discussion

Cancer and some infectious/inflammatory diseases lead to MDSC accumulation in peripheral blood and multiple tissue compartments, including BM[1–6]. A higher frequency of MDSCs in a cancer setting is usually associated with a poor prognosis[7, 34]. In this study, we found unexpectedly that BM MDSCs paradoxically decreased after chronic SIV infection compared with healthy controls. This was in sharp contrast to the general increase of MDSCs observed in BM during cancer and other infectious/inflammatory diseases, and also contrary to the MDSC expansion in HIV/SIV-infected PBMCs [12, 22, 23]. We further demonstrated that even though the changes were among both CD14^+^ and Lin^-^CD15^+^MDSC subsets in BM, and both subsets correlated with high plasma vRNA level and CD4^+^ T cell loss, it was only the reduction of CD14^+^ MDSC that correlated with the high levels of CD8^+^ T cell activation. These changes suggest that the loss of BM MDSCs, especially the CD14^+^ subset, was associated with the progression to HIV/AIDS disease. We then investigated the alteration of MDSCs in the BM of a less pathogenic SHIV-macaque model, in which the animals had moderate plasma vRNA levels at the first 6 weeks of infection, but spontaneously controlled the viral replication after several months. These animals had no signs of immune activation, and did not develop AIDS-related disease. Indeed, in these animals, we found that both subsets of BM MDSCs were unchanged 11 months post-infection. This further supported the association of MDSC decrease in BM with HIV/AIDS disease progression.

During HIV/SIV infection, MDSCs are thought to contribute to immune-pathogenesis by dampening protective immunity through the direct inhibition of T cell function, especially the viral-specific CD8^+^ T cell responses, and thus are deleterious[12, 22, 23]. However, our data suggest more complex and somehow paradoxical roles of MDSCs in HIV/SIV infections. MDSCs suppress anti-viral specific immune responses, which hindered the control of HIV replication [22, 23]. However, MDSCs also have the capacity to antagonize immune activation, which plays a key role in driving viral transmission and replication and many of the disease processes associated with AIDS [46–48]. Consistent with this, the CD14^+^ MDSCs in the BM inversely correlated with immune activation (Ki67^+^CD8^+^T cells) of multiple tissue compartments, suggesting that depletion of BM MDSCs contributes to the immune activation in chronically infected macaques. During late HIV infection, the balance of immune suppression and immune activation tips in favor of the latter due to factors such as gut microbe translocations[49, 50]. It has been proposed that MDSCs are responsible for restoring the balance through various mechanisms, including arginase-1, inducible nitric oxide synthase, reactive oxygen species, and induction of regulatory T cells [46, 51–53]. However, the reduction of MDSCs in the BM may impair this function, and contribute to the relentless immune activation in HIV/SIV chronically infected patients/animals.

We are puzzled by the fact that the MDSC reduction in BM correlated with the CD8^+^ T cell proliferation in not only the MDSC-high tissues but also the MDSC-low tissues. MDSCs did not play a direct role in inhibiting CD8^+^ T cells proliferation in situ in the tissues with low MDSC frequencies, whereas in the MDSC-high compartments, MDSCs could inhibit the proliferation of CD8^+^ T cells efficiently. When the CD8^+^ T cells are equilibrated well among different tissue compartments in the SIV-infected animals, MDSCs suppressing T cell in high-MDSC tissues could affect the levels of T cells in the MDSC-low tissues indirectly when they re-equilibrate. MDSCs could play an important role in suppressing immune activation during HIV infection. Another suppressive mechanism of MDSCs could be the induction of CD4^+^ Treg cells, which could travel to other MDSC-low compartments, and suppress immune responses locally[12–14]. Furthermore, MDSCs induced a type 2 polarization of macrophages in tissues, which were functionally inhibitory [54, 55].

It is worth mentioning that even though both subsets of MDSCs were decreased in the chronically SIV-infected BM, and associated with markers of disease progression: high plasma vRNA levels, low CD4^+^ T cell counts, only loss of CD14^+^ MDSCs was associated with high immune activation. Thus CD14^+^ MDSC and Lin^-^ CD15^+^ MDSC were not only phenotypically distinct, but also played different roles in SIV infections. In cancer settings, CD14^+^ MDSCs have been shown to be the dominant suppressive populations of MDSCs, and correlated with cancer incidence, whereas G-MDSC did not correlate with clinical outcomes, even though both subsets were increased[21]. We also observed more dramatic changes of CD14^+^ MDSC subsets in both PBMC and BM after SIV infection. This implied that the two subsets of MDSCs were under different regulatory mechanisms, and CD14^+^ MDSCs might be the main subset that respond to SIV infection, and determine the outcome of SIV infections. However, as cryopreservation changes the PMN-MDSC (Lin^-^CD15^+^) numbers and function[37, 55], and all the samples used in this study were cryopreserved ones, we should be cautious on the interpretation of data on this subset.

In the present study, to investigate the mechanisms by which the MDSCs were decreased in the SIV-infected BM, we found that infection by virus, migration out of BM, and/or poor self-replenishment of MDSCs might all play important roles. With the data we have, it is impossible to dissect which were more critical. It has been reported that infectious/inactivated HIV, or gp120/gp41, and tat proteins directly promoted MDSC expansion in an *in vitro* culture system using healthy PBMCs [13, 14, 22]. However, we doubt that virus/viral proteins were the key contributors to the MDSC accumulation in the PBMCs of the infected macaques based on 1) dramatic increase of M-MDSCs in the PBMCs of HIV-1-infected patients on antiretroviral therapy with undetectable viremia [14]; 2) no correlations of VLs with MDSC accumulation in PBMC at different time-points in the same animal[23]; and 3) decreased rather than increased MDSCs in BM during chronic SIV infection.

Consistent with the finding of CD14^+^ MDSC susceptibility to HIV-1 infection *in vitro* [22], we further demonstrated by three independent mutually corroborative experimental criteria (gag expression relative to CD3 and CD4 expression, spliced vRNA expression, and p27 protein production) that both subsets of MDSCs were able to be infected *in vivo*. In contrast to the suggestion that the infection of myeloid cells might cause developmental defects in myeloid cells, and result in accumulation of MDSCs in the *in vitro* study [22], our data suggest that infection of MDSCs by SIV *in vivo* might eventually lead to MDSC depletion directly or indirectly. In the SIV-infected PBMC and BM, the SIVgag^+^ MDSCs were a small fraction of the total MDSCs. If infection itself led to MDSC expansion, we would expect a big pool of SIVgag^+^ MDSCs in the infected animals. We noticed that the relative expression level of SIVgag mRNA per cell was comparable between CD4^+^ T cells and MDSCs (Fig 5C), which suggested that MDSCs could be productively infected by SIV, like their CD4^+^ T cell counterparts. Productive infection was confirmed both by detection of spliced SIV mRNA (S7 Fig), and by staining for expression of viral gag protein (Fig 6), under conditions in which contamination with T cells could be ruled out as an explanation. If MDSCs are productively infected with SIV, viral-induced depletion might play an important role. However, further evidence is needed to confirm whether SIV infection leads to MDSC death.

HIV-1 infected patients usually develop hematopoietic dysfunction, which is mainly mediated by the altered production of stromal cell-derived hematopoietic growth factors, infection of the BM accessory cells, and impaired stromal functionality[41–43, 56]. This abnormality could lead to defects of MDSC replenishment. Indeed, we found decreased proliferation (Ki67^+^%) of both subsets of MDSCs in BM after SIV infection.

The inverse or reciprocal relationship of MDSCs in BM and PBMC, e.g., increased in PBMC and decreased in BM, promoted us to investigate the possibility of MDSC redistribution during SIV infection. We compared the chemotactic activities of plasma from the pre- and post-SIV infection blood using *in vitro* chemotaxis assays. It turned out that the chemotaxis index of MDSCs, but not of T cells or total cells, was increased during chronic SIV infection. We believe that this could directly lead to MDSC loss in BM, as a consequence of more MDSCs’ migrating out of the BM. The preferential trafficking of MDSCs towards the post-infection plasma suggested that certain chemokine(s)/cytokine(s) in the chronic SIV infected plasma could preferentially pull the MDSCs out of BM. In the attempt to dissect which chemokine(s)/cytokine(s) were responsible, we tested CCL2, known to affect myeloid cell migration. We found that CCL2 correlated with CD14^+^ MDSC accumulation in the PBMC, but did not correlate with MDSC chemotaxis index measured *in vitro*. Since CCL2 was one of multiple cytokines/chemokines induced by SIV, other chemokines/cytokines might also play roles in attracting MDSCs to the circulation. Antibody blocking for CCL2 would be a good way to assess the role of CCL2 played in chemotaxis. Nevertheless, as MDSCs are such heterogeneous populations and no single chemokine receptor is truly specific for MDSCs, the net chemoattractant effects of multiple chemokines in the plasma might account for the results of the *in vitro* assays.

One caveat was that this was a cross-sectional study of MDSC alteration in BM during the chronic infection stage. A longitudinal study with multiple time-points during acute and chronic infection stages will give us a better picture of the MDSC dynamics, and better understanding of the roles of MDSCs during SIV infection. We speculate that there is a delicate balance of immunity and suppression established at the onset of the SIV infection. During the early stage of the pathogenic SIV infection, it is possible that the MDSC increase/expansion also occurred in BM, as we have observed in the SHIV-infected macaques, in which MDSCs initially significantly increased 4 months post-infection (S10 Fig), and then reverted to normal. In the pathogenic SIV infections, however, the presence of persistent high viral loads, and the poor self-replenishment by MDSC proliferation due to bone marrow stromal functional defects could lead to the gradual decrease of MDSCs in BM. Future studies on the kinetics of MDSCs in the BM of pathogenic SIV infections will clarify this. This depletion of MDSCs eliminates one of the body’s key mechanisms to limit tissue damage from aberrant immune activation. Thus, in the setting of acute SIV infection, MDSCs can limit antiviral T cell responses, whereas in chronic infection, BM MDSC depletion can contribute to the disease progression. Overall, MDSCs act as a double-edged sword in HIV/SIV-infection, and the decrease of MDSCs in BM after SIV infection could serve as an indicator of immune regulatory exhaustion. Further elucidating the roles of MDSCs in HIV/SIV infection will contribute to our understanding of the immune-pathogenesis of HIV infections and contribute to the development of therapeutic approaches for HIV/AIDS.

## Materials and methods

### 1. Ethics statement

All the adult Indian rhesus macaques (*Macaca mulatta*) were used with the approval of Institutional Animal Care and Use Committees. The macaques were housed at the NCI Animal Facility, Bethesda, MD (Protocols No. VB010 and VB011 approved by the NCI IACUC), which is accredited by the Association for Assessment and Accreditation of Laboratory Animal Care (AAALAC) International. The housing and the standard practices closely followed the recommendations of the Standards and Guide for the Care and Use of Laboratory Animals of the United States—National Institutes of Health. All efforts including provision of peri-operative and post-operative analgesia were made to minimize discomfort of the animals. Details of animal welfare, including housing, feeding, environmental enrichment, and steps to minimize suffering, were in accordance with the Guide and the recommendations of the Weatherall report, ‘‘The use of non-human primates in research”, as approved by the IACUCs. Macaques were housed in temperature controlled facilities with temperature of 21–26°C, humidity of 30%– 70%, and a 12 h light/dark cycle. A commercial primate diet and fresh fruit were provided twice daily with water freely available at all times. Macaques were monitored twice daily for overall health including activity, food and water intake. The macaques were singly housed in stainless steel wire-bottomed cages due to the nature of the experiment. Rotating toys, visual and auditory stimuli, and foraging opportunities were provided daily. The animals were anesthetized with approximately 10 mg/kg of ketamine hydrochloride injected intramuscularly for blood and bone marrow collections. When IACUC defined endpoints were reached, macaques were humanely euthanized with an overdose of barbiturate in accordance with the recommendations of the most recent American Veterinary Medical Association Panel on Euthanasia.

### 2. Animals, viral challenge, and viral load (VL) measurements

The 12 simian immunodeficiency viruses (SIV) mac251-infected animals have been described in our previous studies [23, 57] (S1 Table, cohort 1A). Briefly, the animals were intrarectally challenged by three serial SIVmac251 viruses at 2-week intervals until they have been infected. The SIVmac251 viral stock was provided by Nancy Miller of the National Institute of Allergy and Infectious Diseases (NIAID). Twelve simian–human immunodeficiency virus (SHIV)-infected macaques were also included in this study (6 males and 6 females; age: 3,3±1.3 years; weight: 4.6±1.7 kg) (S1 Table, cohort 2A). The animals were intrarectally exposed to **8** serial SHIVSF162P4 viral challenges with a week of interval (1:35 dilution) until they were infected. After the viral challenges, SIV/SHIV RNA levels were monitored by Advanced BioScience Laboratories, Inc. The cut-off threshold for viral RNA detection was 50 copies/ml. Blood and tissue samples from naïve macaques were collected and included in the study for comparisons (S1 Table, cohort 1, 2, 3 and other). Liver tissues from 8 naive (4 adenovirus-exposed, 4 non-exposed, all SIV/SIHV-negative) macaques were used as normal controls (S1 Table, other). Other naïve macaques which have been enrolled in this study were described in S1 Table. Additional SIVmac251 infected bone marrow samples were obtained from California National Primate Research Center (S1 Table).

### 3. Preparation of single-cell suspensions from the tissues

Single-cell suspensions from blood, bone marrow, lymph nodes, and spleens were prepared as previously described [58]. We have used the same protocol to process bone marrow and blood samples. Briefly, the samples were first 1:1 diluted with 1XPBS or HBSS, and then the diluted samples were overlaid on lymphocyte separation medium Lympholyte-H (Human, Cedarlane, Ontario, Canada). After centrifugation at 800 x g for 20 min at room temperature (25°C), with centrifuge brake on OFF position, the middle opaque fluid containing the PBMC/BM cells was collected. The cells were washed with R10 medium three times before cryopreservation in liquid nitrogen freezers. Ileum lamina propria, colon lamina propria, and liver were collected from the macaques as previously described with modification[23, 57–59]. Briefly, the tissues were first rinsed 2–3 times with 1XHBSS containing 5mM EDTA and 1-2mM DTT to remove mucous and intestinal intraepithelial lymphocytes (IEL) (only for the gut). The tissues were then cut into small pieces and incubated with Liberase (Roche) at 37°C for 30 min. After enzyme digestion, the tissue chunks were mashed through a syringe end and filtered through a 100 μm cell strainer. After washes, the cells were re-suspended in 40% Percoll, and underlaid with 80% Percoll. The cells in the interface were collected after spinning down for 20min, 800 x g at room temperature (25°C), with centrifuge brake on OFF position. The cells were cryopreserved in liquid nitrogen freezers after three washes with R10 medium. All cell samples used in this study were cryopreserved.

### 4. Antibodies and flow cytometric analysis of MDSC and immune activation of the tissue compartments

For flow cytometric analysis, the single-cell suspensions from different compartments were first incubated with Fc Receptor blocking reagent (Miltenyi Biotec), and then stained with viability dye (Invitrogen) and antibody mixtures including anti-CD45 (BD Pharmingen) to exclude the dead and CD45 negative cells. For immune activation, the following antibodies were used: CD3-PE-Cy7, CD4-BV605, CD8-APC-Cy7, CD14-V450, Ki67-APC, HLA-DR PE-Cy5, and CCR5-PE (BD Pharmingen); CD69-Alexa Fluor 700 (Biolegend); and CD38-FITC (STEMCELL Technologies). For MDSC analysis, the following antibodies were used: CD3-PE-Cy7, CD4-BV605, CD14-V450, Ki67-APC, HLA-DR-APC-Cy7, Lin-FITC (Lin1, BD Pharmingen); CD33-PE (Miltenyi Biotec), CD15-Alexa700 and CD11b-PE-Cy5 (Biolegend). CM9 Dextramer was obtained from Immudex (Denmark). For gag-P27 staining of MDSCs, besides the antibodies listed above, CD2-PE-Cy7 (BD Pharmingen), CD68-Perp-cy5.5, and P27 antibody [60] (clone KK64, cat#2321, from NIH AIDS reagent program) were also added to the panel. For gag-P27 staining, we have incubated the cells for 2 hrs with 5 μl of human TruX blocker (Biolegend), and 2 μl of human IgG (1 mg/ml, from R&D systems) before adding the surface antibody mixture. We also extended the P27 staining overnight on ice. An LSRII flow cytometer was used for data acquisition, and FlowJo software (Tree Star Inc.) was used for data analyses. The gating strategies for MDSCs were shown in Fig 1A and Fig 2A.

### 5. Flow cytometric sorting and SIV viral RNA detection

MDSCs were sorted by using a BD FACSAria (BD Biosciences) with the following markers: CD3^–^HLA-DR^–^CD11b^+^CD33^+^ cells from live PBMCs (purity greater than 95%) or spleen. CD4^+^ T cells were sorted as positive controls. The sorted CD4^+^ T cells were either serially diluted in 10-fold dilutions or directly put into Trizon. After Trizon-lysis, the sorted cells were then subjected to RNA isolation, reverse transcription, and qPCR reactions to detect the relative expression levels of SIVgag, spliced SIV rev, tat, nef, and vif, CD3, CD4, and GAPDH in MDSCs and CD4^+^ T cells (ABI, Bioline) [58]. Taqman probe and primer sets for gag[61], CD3, CD4, and GAPDH were used (ABI). Spliced SIV rev, tat, nef, and vif primers and probes were synthesized at ABI, and multiplexed to detect the rev, tat, nef, and vif targets simultaneously [62]. Each PCR mixture contained 5 μl of cDNA, 10 μl of 2X universal PCR master mix (ABI) or SensiFast probe kit (Bioline USA Inc.), 1 μl of primer/probe set. All PCR were run using ABI 7500 with program consisting of 2 min at 50°C, 10 min at 95°C, and then followed by 45 cycles of 15 sec at 95° C, and 1 min at 60°C. Relative mRNA expression levels were compared by the comparative threshold cycle (Ct) method of relative quantitation (PerkinElmer User Bulletin no. 2) as described[63], except in cases where standards were available to determine copy number, as indicated. As each cycle represents a doubling (log_2_), subtracting Ct for GAPDH is essentially taking a ratio to or normalizing to the GAPDH housekeeping gene.

### 6. Chemotaxis assays

The 96-well ChemoTx chemotaxis system (NeuroProbe Inc., 5um pore) was used for chemotaxis assays. The lower wells were blocked with 301 μl of 1% BSA for 30 min at room temperature, which was aspirated and replaced with 301 μl of plasma from pre- or post-infected macaques as test samples. 301 μl of RPMI-1640 /0.1% BSA buffer was included in the experiments as buffer controls. Single cell suspensions from bone marrow of naive macaques were collected and 2 x 10^5^ bone marrow single cell suspensions were re-suspended in 50 μl RPMI-1640 /0.1% BSA and loaded above the membrane. After incubation for 2 hr at 37°C, 5% CO_2_, the top wells were removed with a scraper and the migrated cells in the bottom wells were counted, and stained with the antibody mixture: CD3-PE-Cy7, CD14-V450, HLA-DR-APC-Cy7, Lin-FITC, CD33-PE, and CD11b-PE-Cy5, Cd45-Alexa 700, and yellow viability dye. 50 μl of CountBright absolute counting beads (Molecular Probes) were added to each tube before data acquisition using an LSRII flow cytometer, and FlowJo software (Tree Star Inc.) was used for data analyses. Chemotaxis index was defined as the ratio of the absolute cell numbers in the test samples over the absolute cell numbers in the buffer control samples.

### 7. Statistical analyses

We performed statistical analyses with Prism version 6 (Graph Pad). One-way ANOVA with Dunn’s multiple comparison corrections, and Mann-Whitney, and Wilcoxon tests were used as shown in the figures. Spearman analysis was used for correlations. A two-sided significance level of 0.05 was used for all analyses.

## Supporting information

S1 Fig Gating strategy of CD14^high^ and CD14 ^intermediate^ MDSCs in SIV chronically infected bone marrow (14-month post-infection) and naïve bone marrow. (TIF)

S2 Fig CD14^+^ subpopulation in SIV-infected and naive bone marrows. CD14^+^ subpopulation within the CD45^+^CD3^-^ live mononuclear cell gate from two cohorts of naïve macaques (with 28 and 12 animals separately), and one cohort of SIV chronically infected macaques (12 animals) were shown.
(TIF)

S3 Fig The frequencies of CD14^+^ and Lin^-^CD15^+^MDSC subsets in freshly-isolated vs. cryopreserved/thawed bone marrow (BM) and PBMC samples were compared. BM and PBMC samples from SIVmac251-infected and naïve macaques were divided into two aliquots; one was immediately stained and analyzed by flow-cytometry, while the other were cryopreserved in liquid N_2_ and thawed for testing 4–5 months later. SIV-infected BM (n = 6), PBMC(n = 7), and naïve BM (n = 12), PBMC (n = 5) samples were used for comparisons. (A-B) The frequencies of CD14^+^MDSCs in the BM samples did not significantly change in the SIV-infected and naïve animals, where the frequencies of Lin^-^CD15^+^MDSCs significantly decreased after cryopreservation and thawing. (C) 70–80% of CD14^+^MDSCs were maintained in the frozen naïve and SIV-infected BM samples, whereas only 20% of Lin^-^CD15^+^MDSCs were detected after cryopreservation and thawing in both the infected and naïve animals. No difference in preservation was observed between SIV-infected and naïve animals for either subset. (D-F) The frequencies of CD14^+^MDSCs in the PBMC samples did not significantly change in the SIV-infected animals after cryopreservation and thawing. The frequencies of Lin^-^CD15^+^ and CD14^+^MDSCs in the PBMC of the naïve animals, and the frequencies of Lin^-^CD15^+^ MDSCs in the PBMC of SIV-infected animals were too low to adequately assess the effect on cryopreservation, although the direction of the change in CD15^+^ Lin^-^ MDSCs in PMBC (Panel E) was in the direction of greater preservation in the SIV-infected than in the naïve populations, not consistent with any greater loss in the SIV-infected cells.
Each data point represents one individual animal. The Wilcoxon matched-pairs signed rank tests were used for comparisons.
(TIFF)

S4 Fig The frequencies of CD14^intermediate^ and CD14^high^ MDSCs in the bone marrow of chronically SIV-infected macaques inversely correlated with plasma viral loads; but only CD14^intermediate^, but not CD14^high^, MDSCs positively correlated with CD4^+^ T cell preservation in the PBMC. Spearman analysis was used for correlations.
(TIF)

S5 Fig qPCR analysis of SIV-gag in the flow cytometer sorted MDSCs and CD4^+^T cells from the PBMCs of SIV-infected macaques. The Ct values of Gag, CD4, CD3, and GAPDH from the sorted MDSCs and CD4^+^T cells were shown in A&B. Equal amounts of cDNA were used to detect the expression levels of gag, CD4, CD3, and GAPDH except the CD3 in the sorted CD4^+^T cells (100-fold less cDNA was added) using macaque-specific Taqman primer/probes. The ratio of SIVgag /CD4 in CD4^+^T cells and MDSCs were shown in C.
(TIFF)

S6 Fig qPCR analysis of SIV-gag in the flow cytometer sorted MDSCs and CD4^+^T cells from the PBMCs of SIV-infected macaques. The Ct values of Gag, CD4, and GAPDH from the sorted MDSCs and CD4^+^T cells were shown in A-C. Equal amounts of cDNA were used to detect the expression levels of gag, and CD4 using macaque-specific Taqman primer/probes. The ratio of SIVgag expression level in MDSCs vs SIV gag expression level in CD4^+^T cells is shown in D, calculating using 2^ (Δgag-GAPDH) for MDSCs vs CD4^+^T cells. The ratio of CD4 expression level in MDSCs vs CD4 expression level in CD4^+^T cells were shown also in D using the similar calculation method. MDSCs had a 50-fold lower expression level of CD4. Thus, the level of CD4^+^ T cell contamination in the MDSC population is 50-fold too low to account for the SIVgag present.
(TIF)

S7 Fig qPCR analysis of SIV mRNA in the flow cytometer sorted MDSCs and CD4^+^ T cells from the PBMC or BM samples of the SIV-infected macaques. Three independent experiments are shown. MDSCs and CD4^+^ T cells from the PBMC or bone marrow samples of the SIVmac251 chronically infected animals were sorted as described in Fig 5A. After RNA isolation and cDNA synthesis, each of the CD4^+^ T and MDSC samples was subjected to qPCR analysis for SIVgag and/or spliced SIV mRNA, CD3, or CD4, and GAPDH using the same amounts of cDNA for that sample. (A-B) In this experiment, four PBMC specimens from the SIVmac251-infected macaques were analyzed for unspliced SIVgag and spliced SIV mRNA. The threshold cycles of spliced and unspliced viral mRNA in T cells and MDSCs of the infected macaques are shown in A-B. Lower threshold cycle value indicates higher expression level of the target sequence. For CD3, if there was no amplification signal after 45 cycles, Ct value of 45 was assigned to the tested sample. (C) In another independent experiment, MDSCs and CD4^+^ T cells from the bone marrow samples of the six SIV-infected animals were sorted. For the sorted CD4^+^ T cells, the samples were serially diluted (10–fold) before the RNA isolation. Spliced SIV mRNA and CD3 mRNA from six MDSC samples and one example of CD4^+^ T cells were analyzed. The detection limit is 90 to 900000 CD4^+^ T cells. Of the 4 animals with no detectable CD3 contamination (shown in magenta squares), 3 had clear spliced mRNA expression. (D) In the third independent experiment, MDSCs and CD4^+^ T cells from the PBMC of six SIV-infected animals were sorted. For the sorted CD4^+^ T cells, the samples were serially diluted (10–fold) before the RNA isolation. Gag and CD4 were shown in Fig 5D. For CD3, if there was no amplification signal after 45 cycles, Ct value of 45 was assigned to the tested sample. Black dots in C are CD4^+^ T cells as positive control titration curve, while red or magenta squares are the MDSCs plotted on the same scale. Black dots in D are CD4^+^ T cells as positive control titration curve, while red squares are the MDSCs plotted on the same scale. The sensitivity to detect T cell contamination in these four animals was from 27 and 118 CD4^+^ T cells.
(TIFF)

S8 Fig Flow cytometric detection of gag-P27^+^ MDSCs and CD4^+^T cells in the bone marrow of SIV-infected or naive animals. Flow cytometric plots of P27 staining (A), and summary of P27^+^ % within CD4^+^T cells, and MDSCs (B) from one experiment with 1 SIV-infected and 4 naïve animals were shown.
(TIF)

S9 Fig Decreased Lin^-^ CD15^+^MDSCs in bone marrow were not associated with increased immune activation except in PBMC. Spearman analysis was used for correlations. The correlations between the frequency of Lin^-^ CD15^+^MDSCs and the Ki67^+^CD8^+^ T cell frequencies in the SIV-infected bone marrow (A), PBMC (B), spleen (D), Axillary LN (E) and Mesenteric LN (F), but not liver (C), ileum LP (G) or colon LP (H) were shown. Each data point represents one animal.
(TIF)

S10 Fig The frequencies of MDSCs were initially significantly increased 4 months post-SHIV_SF162P4_ infection, and reverted to normal 11 months post-infection. (TIF)

S1 Table Information on the macaques used in this study. (PPTX)

S2 Table The R and P values of Spearman’s analysis among the CD14+MDSC from different compartments. (PPTX)

S3 Table The R and P values of Spearman’s analysis among the Lin-CD15+MDSC from different compartments. (PPTX)

S4 Table The R and P values of Spearman’s analysis among the CM9 Dextramer+ frequency within the total CD8+T cells from different compartments. (PPTX)

S5 Table The R and P values of Spearman’s analysis among the Ki67^+^ frequency within the total CD8+T cells from different compartments. (PPTX)
