# Supplementary figures and images for: Paradoxical myeloid-derived suppressor cell reduction in the bone marrow of SIV chronically infected macaques
Source: PLoS Pathog. 2017 May 12;13(5):e1006395. doi: 10.1371/journal.ppat.1006395 (PMC5448820; doi:10.1371/journal.ppat.1006395)

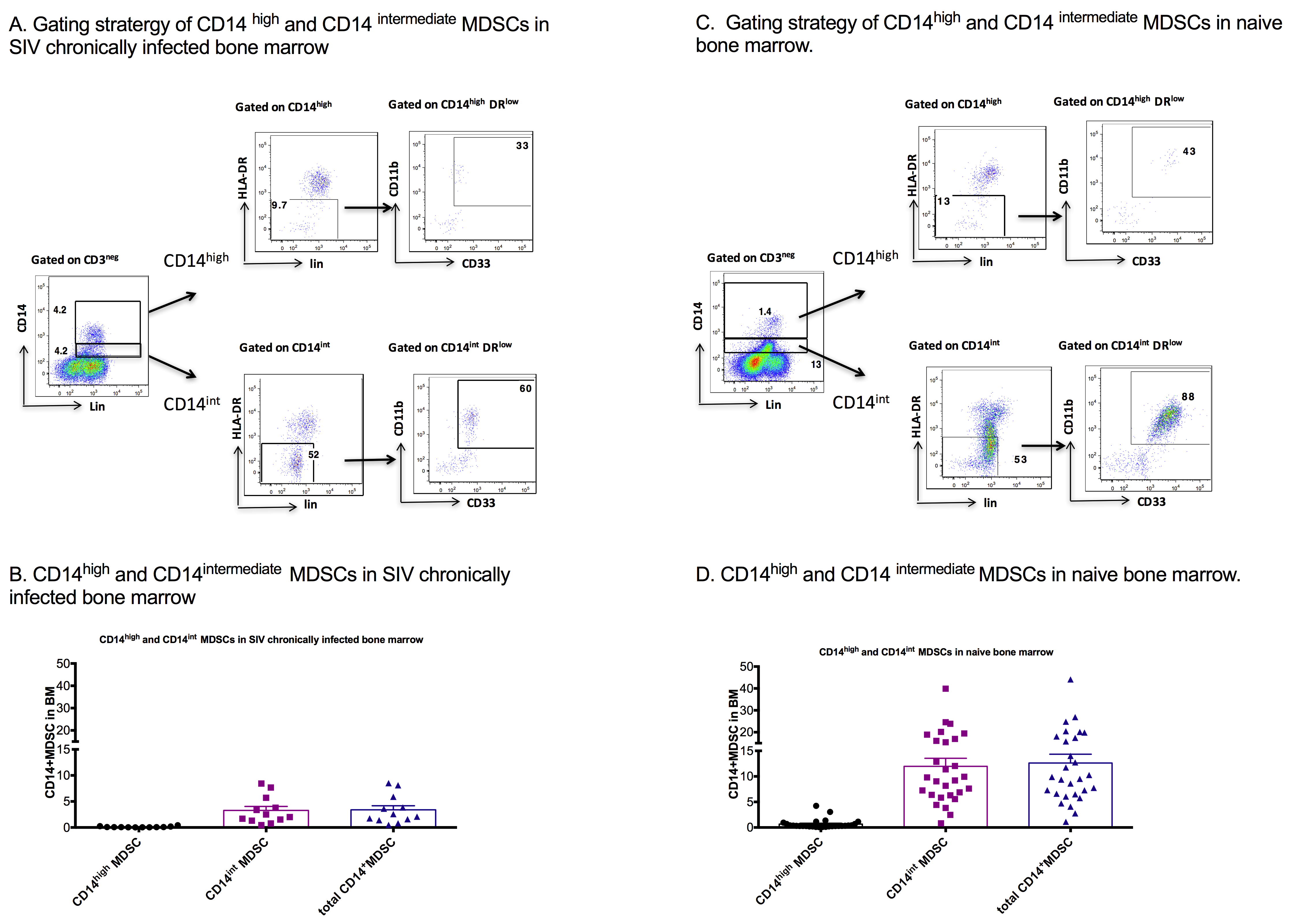

Supplement: S1 Fig — (TIF) [file ppat.1006395.s001.tif]

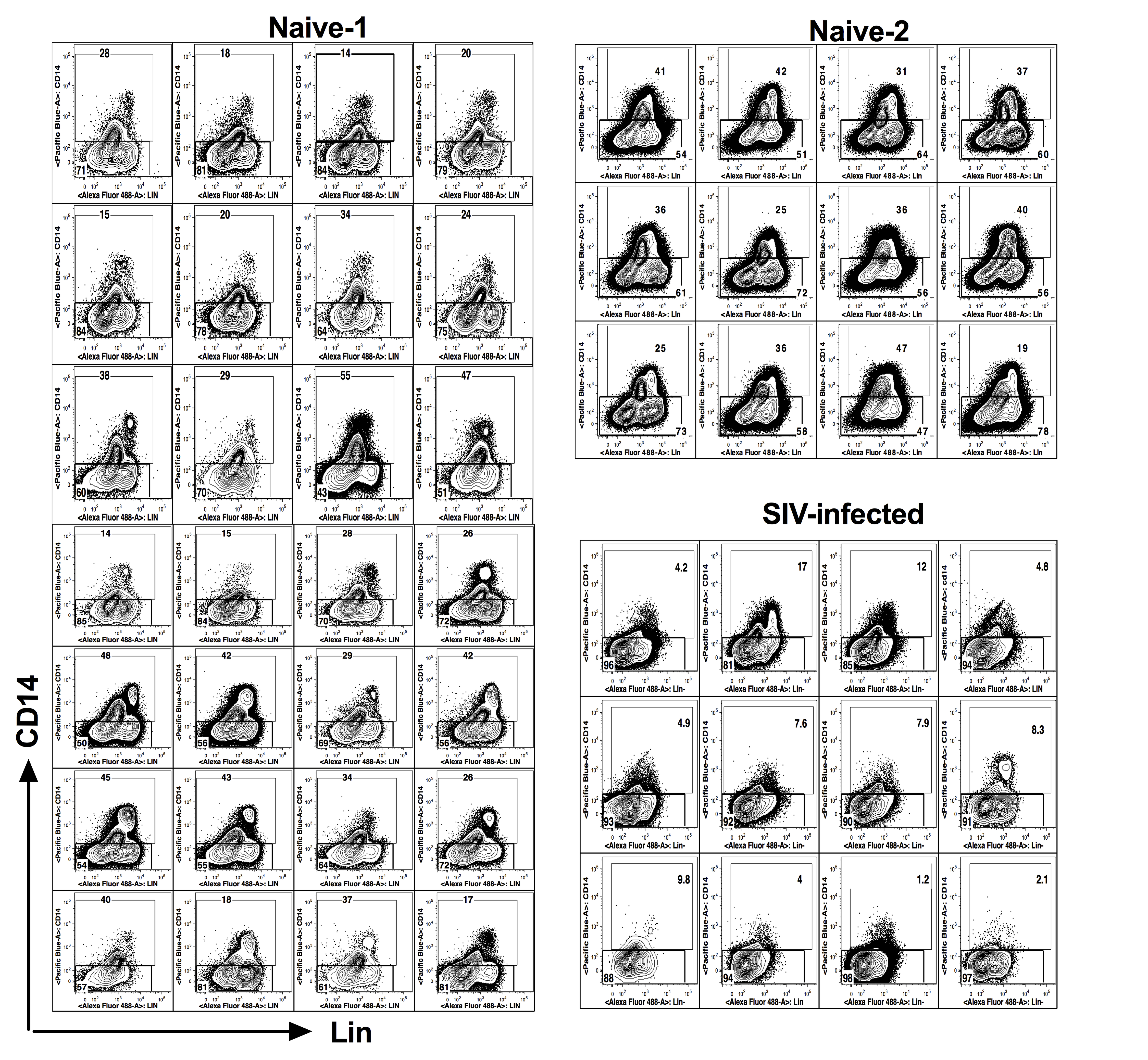

Supplement: S2 Fig — CD14+ subpopulation within the CD45+CD3- live mononuclear cell gate from two cohorts of naïve macaques (with 28 and 12 animals separately), and one cohort of SIV chronically infected macaques (12 animals) were shown. (TIF) [file ppat.1006395.s002.tif]

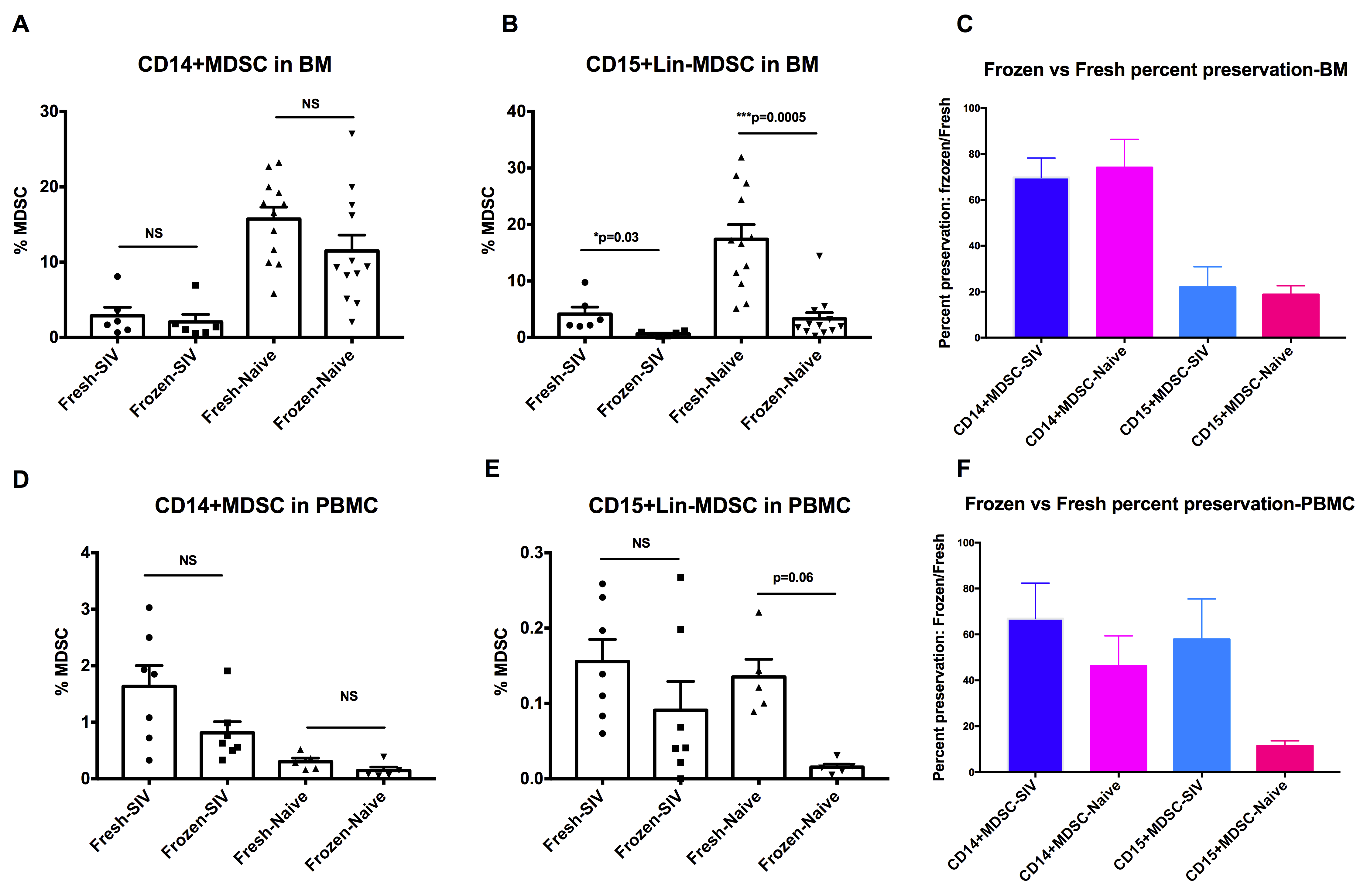

Supplement: S3 Fig — BM and PBMC samples from SIVmac251-infected and naïve macaques were divided into two aliquots; one was immediately stained and analyzed by flow-cytometry, while the other were cryopreserved in liquid N2 and thawed for testing 4–5 months later. SIV-infected BM (n = 6), PBMC(n = 7), and naïve BM (n = 12), PBMC (n = 5) samples were used for comparisons. (A-B) The frequencies of CD14+MDSCs in the BM samples did not significantly change in the SIV-infected and naïve animals, where the frequencies of Lin-CD15+MDSCs significantly decreased after cryopreservation and thawing. (C) 70–80% of CD14+MDSCs were maintained in the frozen naïve and SIV-infected BM samples, whereas only 20% of Lin-CD15+MDSCs were detected after cryopreservation and thawing in both the infected and naïve animals. No difference in preservation was observed between SIV-infected and naïve animals for either subset. (D-F) The frequencies of CD14+MDSCs in the PBMC samples did not significantly change in the SIV-infected animals after cryopreservation and thawing. The frequencies of Lin-CD15+ and CD14+MDSCs in the PBMC of the naïve animals, and the frequencies of Lin-CD15+ MDSCs in the PBMC of SIV-infected animals were too low to adequately assess the effect on cryopreservation, although the direction of the change in CD15+ Lin- MDSCs in PMBC (Panel E) was in the direction of greater preservation in the SIV-infected than in the naïve populations, not consistent with any greater loss in the SIV-infected cells. Each data point represents one individual animal. The Wilcoxon matched-pairs signed rank tests were used for comparisons. (TIFF) [file ppat.1006395.s003.tiff]

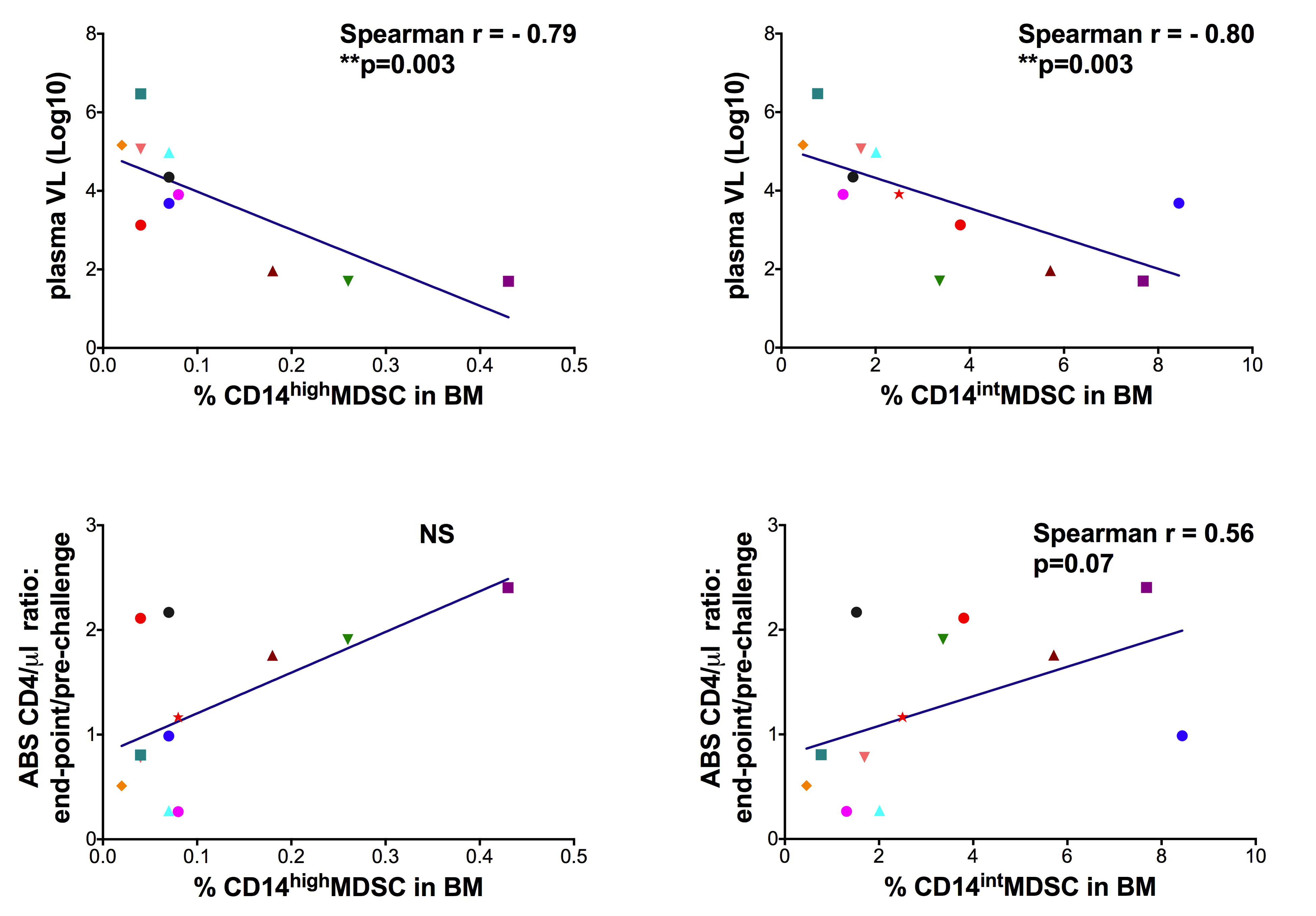

Supplement: S4 Fig — Spearman analysis was used for correlations. (TIF) [file ppat.1006395.s004.tif]

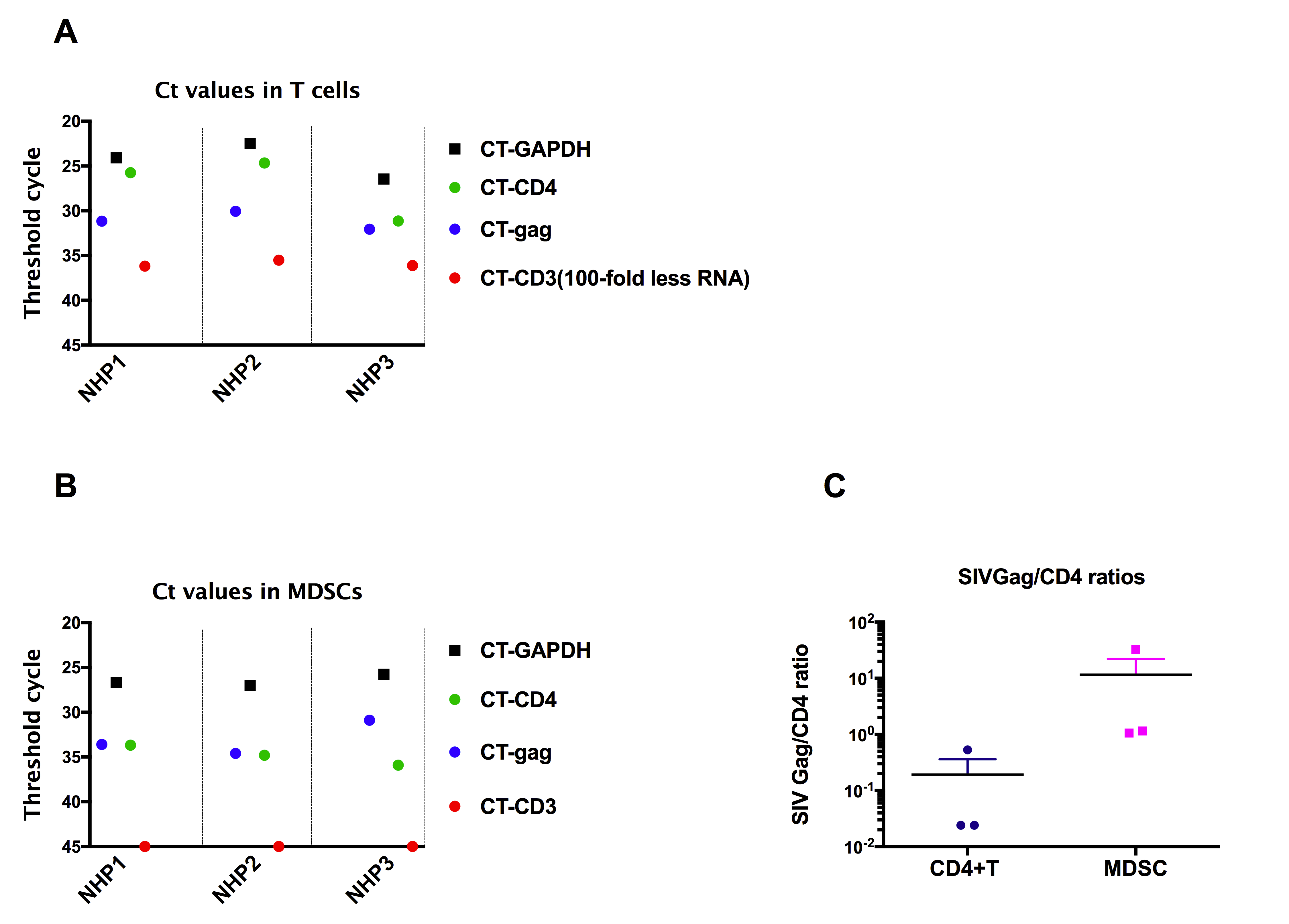

Supplement: S5 Fig — The Ct values of Gag, CD4, CD3, and GAPDH from the sorted MDSCs and CD4+T cells were shown in A&B. Equal amounts of cDNA were used to detect the expression levels of gag, CD4, CD3, and GAPDH except the CD3 in the sorted CD4+T cells (100-fold less cDNA was added) using macaque-specific Taqman primer/probes. The ratio of SIVgag /CD4 in CD4+T cells and MDSCs were shown in C. (TIFF) [file ppat.1006395.s005.tiff]

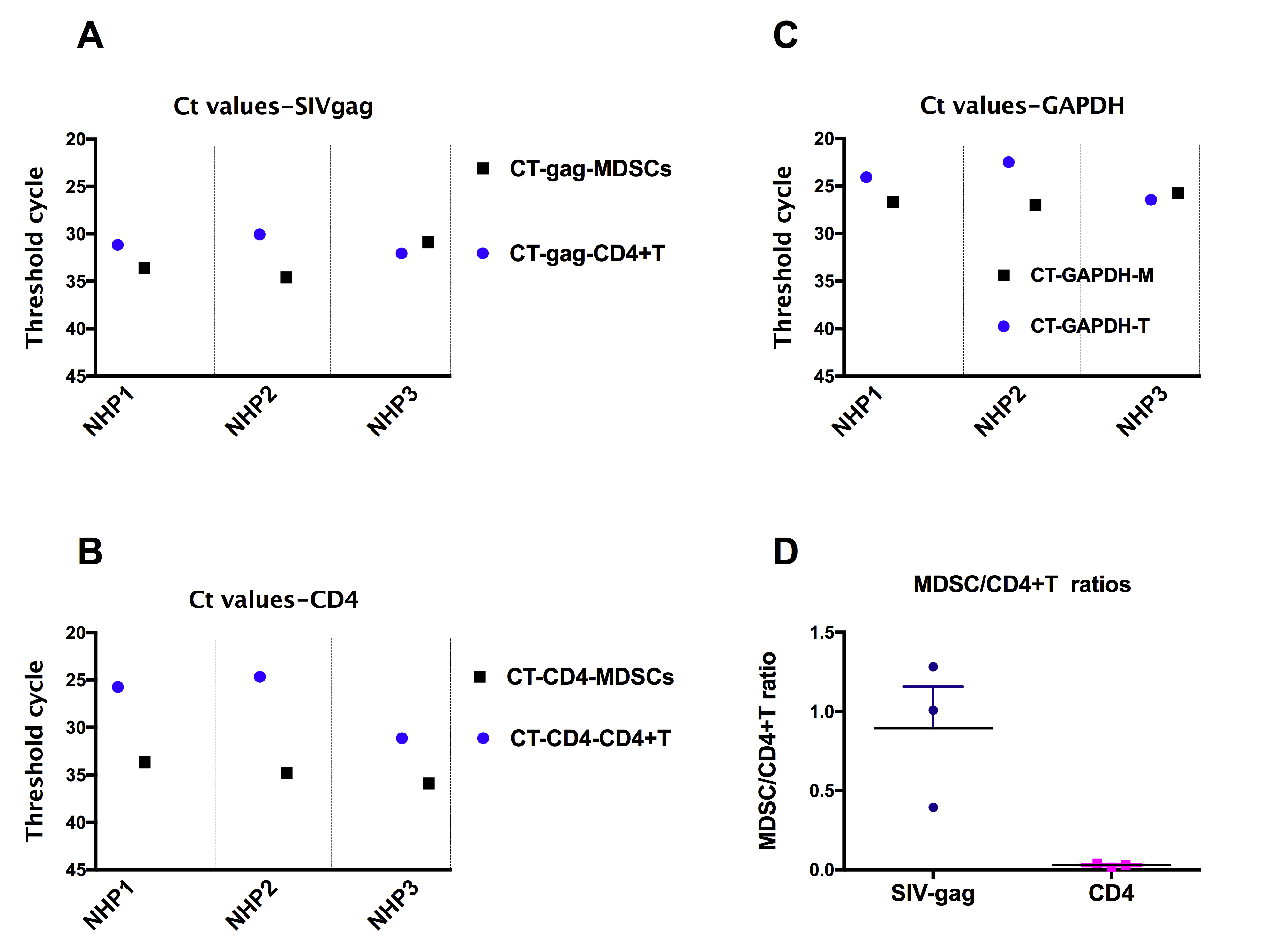

Supplement: S6 Fig — The Ct values of Gag, CD4, and GAPDH from the sorted MDSCs and CD4+T cells were shown in A-C. Equal amounts of cDNA were used to detect the expression levels of gag, and CD4 using macaque-specific Taqman primer/probes. The ratio of SIVgag expression level in MDSCs vs SIV gag expression level in CD4+T cells is shown in D, calculating using 2^ (Δgag-GAPDH) for MDSCs vs CD4+T cells. The ratio of CD4 expression level in MDSCs vs CD4 expression level in CD4+T cells were shown also in D using the similar calculation method. MDSCs had a 50-fold lower expression level of CD4. Thus, the level of CD4+ T cell contamination in the MDSC population is 50-fold too low to account for the SIVgag present. (TIF) [file ppat.1006395.s006.tif]

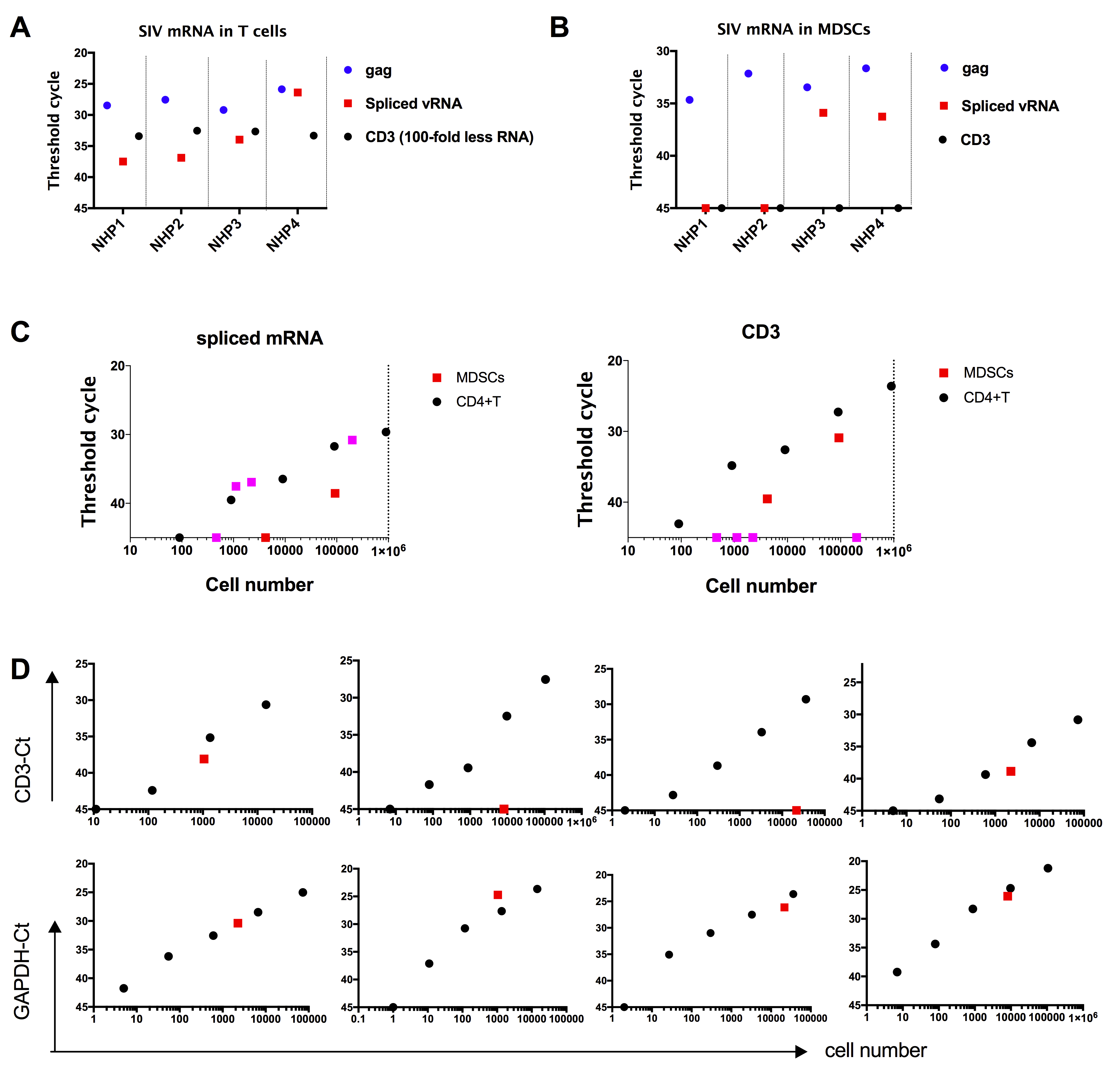

Supplement: S7 Fig — Three independent experiments are shown. MDSCs and CD4+ T cells from the PBMC or bone marrow samples of the SIVmac251 chronically infected animals were sorted as described in Fig 5A. After RNA isolation and cDNA synthesis, each of the CD4+ T and MDSC samples was subjected to qPCR analysis for SIVgag and/or spliced SIV mRNA, CD3, or CD4, and GAPDH using the same amounts of cDNA for that sample. (A-B) In this experiment, four PBMC specimens from the SIVmac251-infected macaques were analyzed for unspliced SIVgag and spliced SIV mRNA. The threshold cycles of spliced and unspliced viral mRNA in T cells and MDSCs of the infected macaques are shown in A-B. Lower threshold cycle value indicates higher expression level of the target sequence. For CD3, if there was no amplification signal after 45 cycles, Ct value of 45 was assigned to the tested sample. (C) In another independent experiment, MDSCs and CD4+ T cells from the bone marrow samples of the six SIV-infected animals were sorted. For the sorted CD4+ T cells, the samples were serially diluted (10–fold) before the RNA isolation. Spliced SIV mRNA and CD3 mRNA from six MDSC samples and one example of CD4+ T cells were analyzed. The detection limit is 90 to 900000 CD4+ T cells. Of the 4 animals with no detectable CD3 contamination (shown in magenta squares), 3 had clear spliced mRNA expression. (D) In the third independent experiment, MDSCs and CD4+ T cells from the PBMC of six SIV-infected animals were sorted. For the sorted CD4+ T cells, the samples were serially diluted (10–fold) before the RNA isolation. Gag and CD4 were shown in Fig 5D. For CD3, if there was no amplification signal after 45 cycles, Ct value of 45 was assigned to the tested sample. Black dots in C are CD4+ T cells as positive control titration curve, while red or magenta squares are the MDSCs plotted on the same scale. Black dots in D are CD4+ T cells as positive control titration curve, while red squares are the MDSCs plotted on the same scale. The se [file ppat.1006395.s007.tiff]

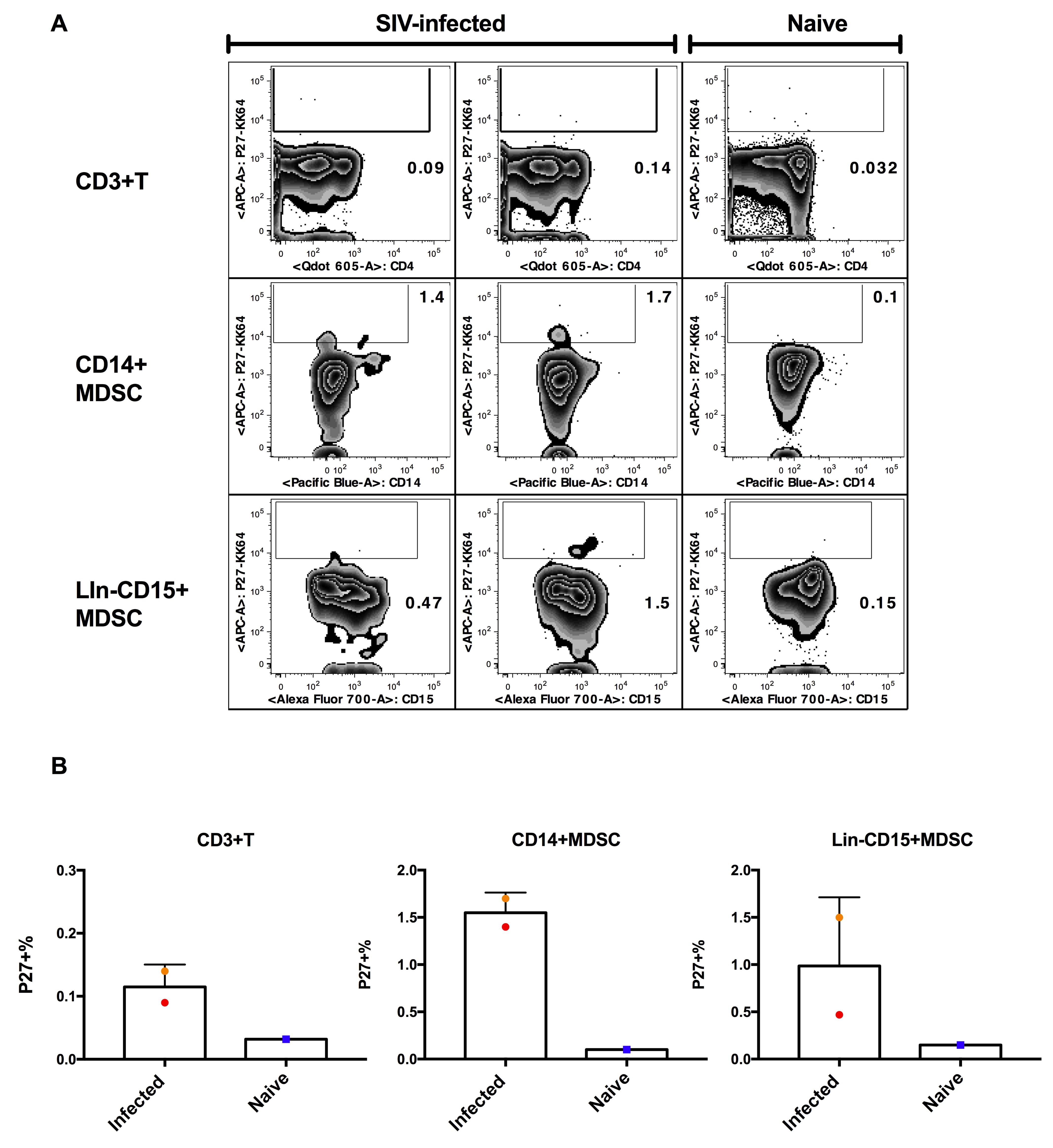

Supplement: S8 Fig — Flow cytometric plots of P27 staining (A), and summary of P27+ % within CD4+T cells, and MDSCs (B) from one experiment with 1 SIV-infected and 4 naïve animals were shown. (TIF) [file ppat.1006395.s008.tif]

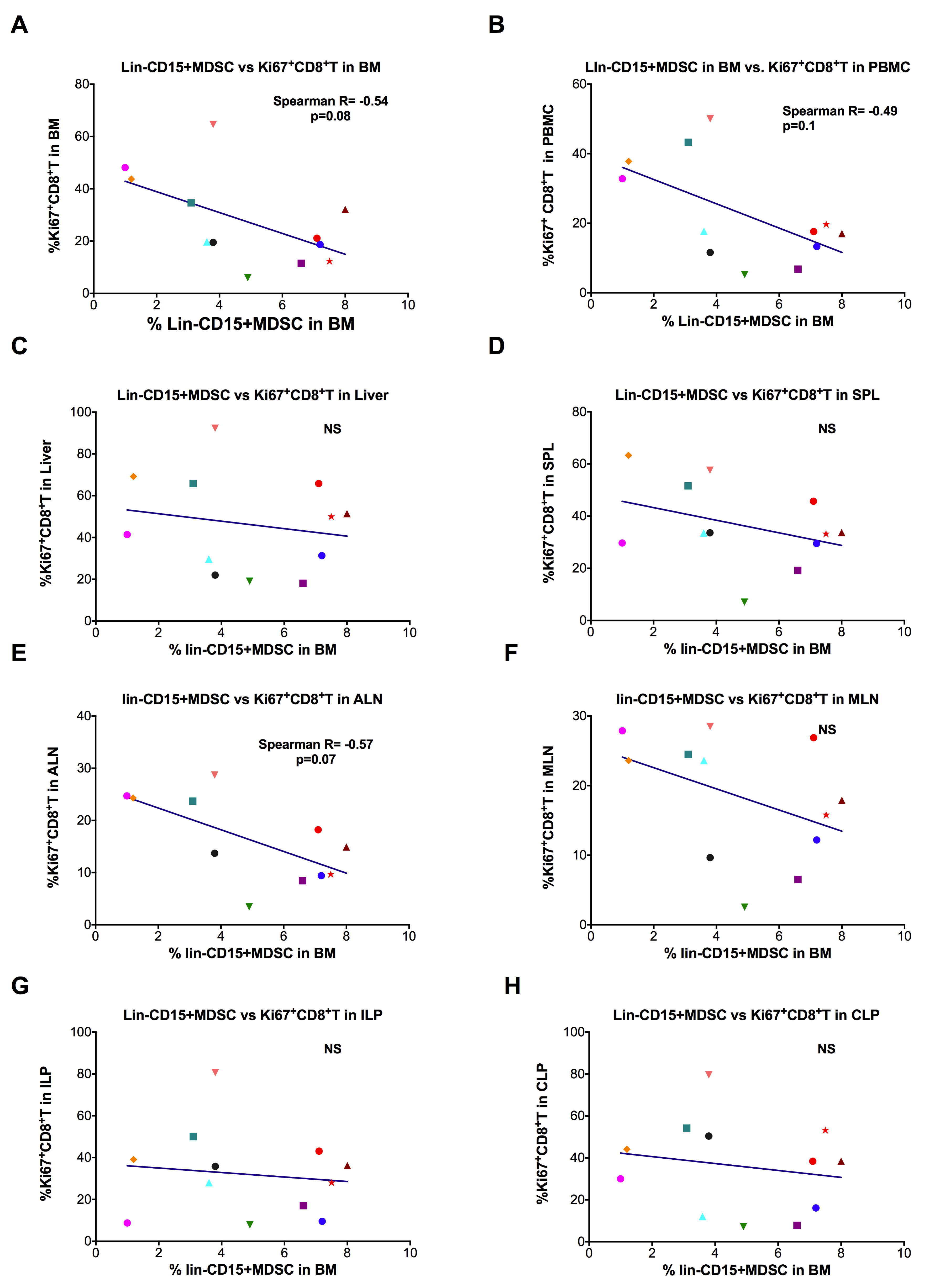

Supplement: S9 Fig — Spearman analysis was used for correlations. The correlations between the frequency of Lin- CD15+MDSCs and the Ki67+CD8+ T cell frequencies in the SIV-infected bone marrow (A), PBMC (B), spleen (D), Axillary LN (E) and Mesenteric LN (F), but not liver (C), ileum LP (G) or colon LP (H) were shown. Each data point represents one animal. (TIF) [file ppat.1006395.s009.tif]

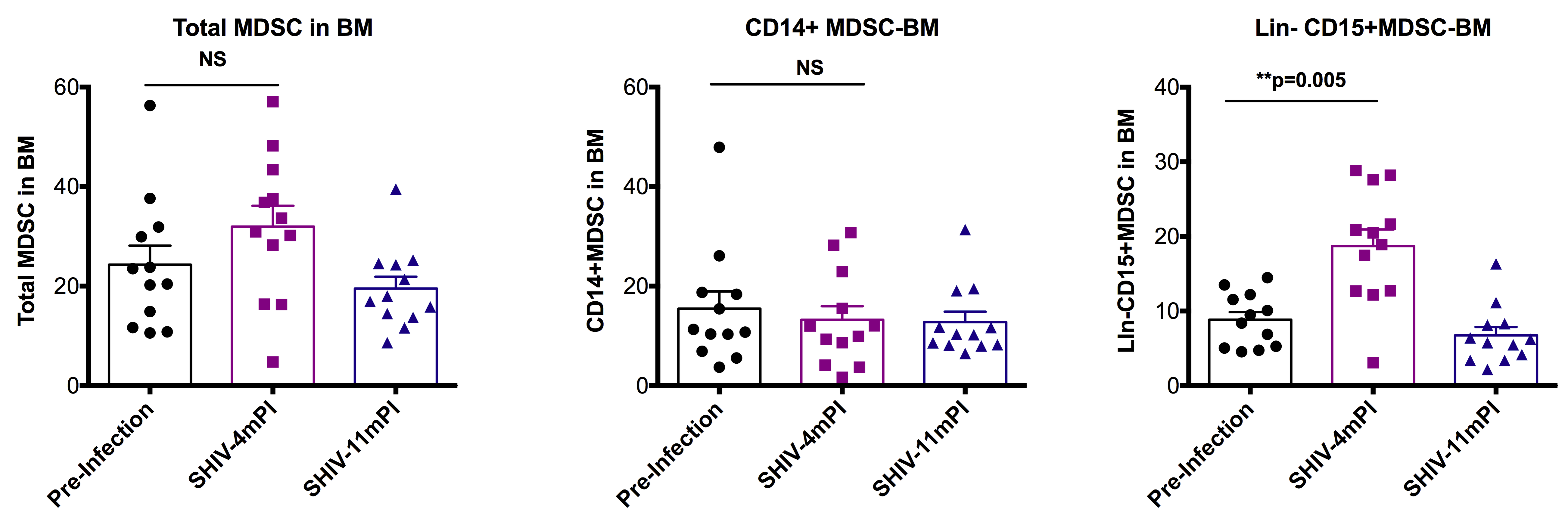

Supplement: S10 Fig — (TIF) [file ppat.1006395.s010.tif]
