# Supplementary material for: Paradoxical myeloid-derived suppressor cell reduction in the bone marrow of SIV chronically infected macaques
Source: PLoS Pathog. 2017 May 12;13(5):e1006395. doi: 10.1371/journal.ppat.1006395 (PMC5448820; doi:10.1371/journal.ppat.1006395)
Supplement: S1 Table — (PPTX) [file ppat.1006395.s011.pptx]

## Slide 1
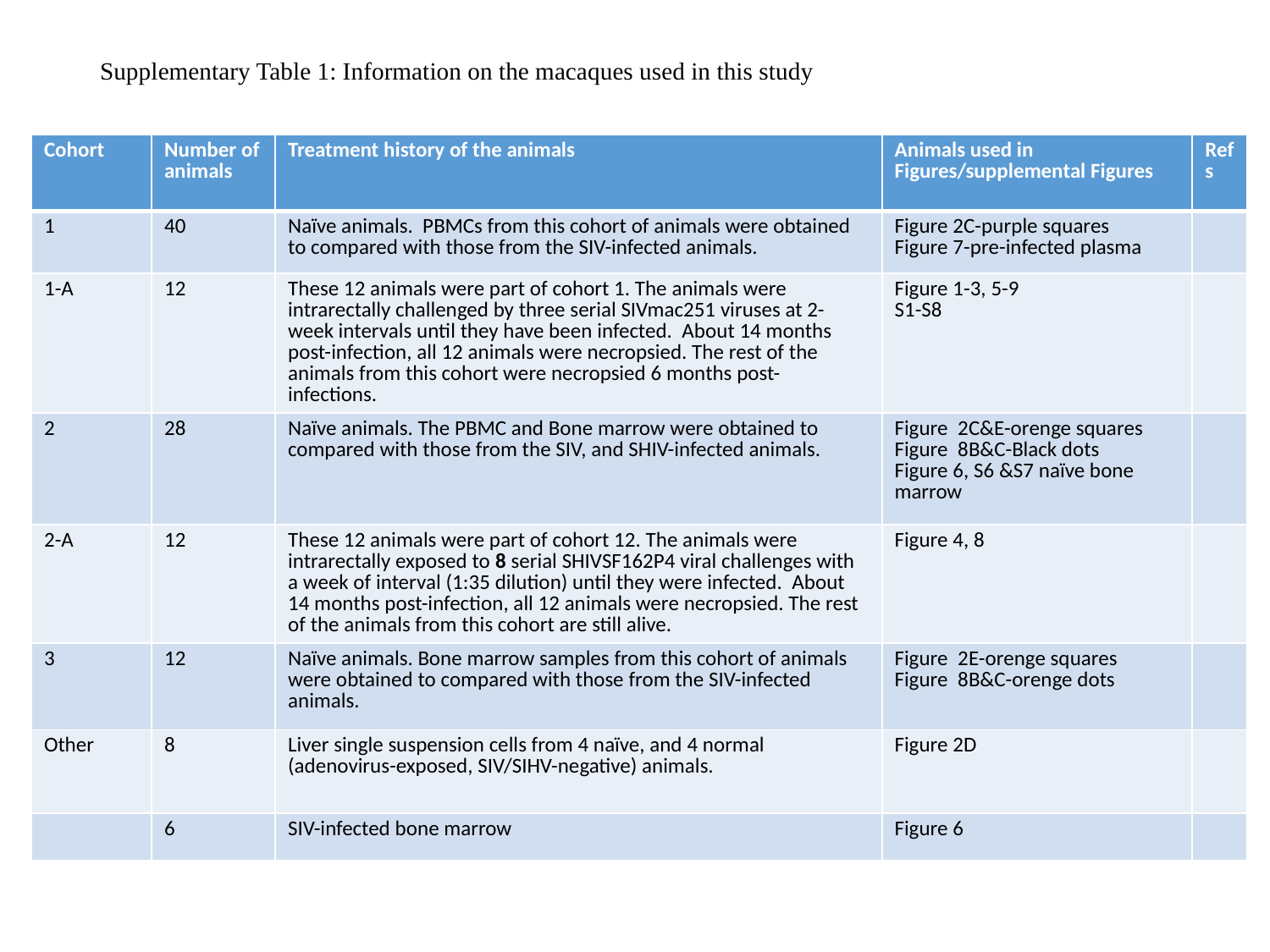

# Supplementary Table 1: Information on the macaques used in this study
| Cohort | Number of animals | Treatment history of the animals | Animals used in Figures/supplemental Figures | Refs |
| --- | --- | --- | --- | --- |
| 1 | 40 | Naïve animals. PBMCs from this cohort of animals were obtained to compared with those from the SIV-infected animals. | Figure 2C-purple squares Figure 7-pre-infected plasma | |
| 1-A | 12 | These 12 animals were part of cohort 1. The animals were intrarectally challenged by three serial SIVmac251 viruses at 2-week intervals until they have been infected. About 14 months post-infection, all 12 animals were necropsied. The rest of the animals from this cohort were necropsied 6 months post-infections. | Figure 1-3, 5-9 S1-S8 | |
| 2 | 28 | Naïve animals. The PBMC and Bone marrow were obtained to compared with those from the SIV, and SHIV-infected animals. | Figure 2C&E-orenge squares Figure 8B&C-Black dots Figure 6, S6 &S7 naïve bone marrow | |
| 2-A | 12 | These 12 animals were part of cohort 12. The animals were intrarectally exposed to 8 serial SHIVSF162P4 viral challenges with a week of interval (1:35 dilution) until they were infected. About 14 months post-infection, all 12 animals were necropsied. The rest of the animals from this cohort are still alive. | Figure 4, 8 | |
| 3 | 12 | Naïve animals. Bone marrow samples from this cohort of animals were obtained to compared with those from the SIV-infected animals. | Figure 2E-orenge squares Figure 8B&C-orenge dots | |
| Other | 8 | Liver single suspension cells from 4 naïve, and 4 normal (adenovirus-exposed, SIV/SIHV-negative) animals. | Figure 2D | |
| | 6 | SIV-infected bone marrow | Figure 6 | |
