# Supplementary material for: Paradoxical myeloid-derived suppressor cell reduction in the bone marrow of SIV chronically infected macaques
Source: PLoS Pathog. 2017 May 12;13(5):e1006395. doi: 10.1371/journal.ppat.1006395 (PMC5448820; doi:10.1371/journal.ppat.1006395)
Supplement: S2 Table — (PPTX) [file ppat.1006395.s012.pptx]

## Slide 1
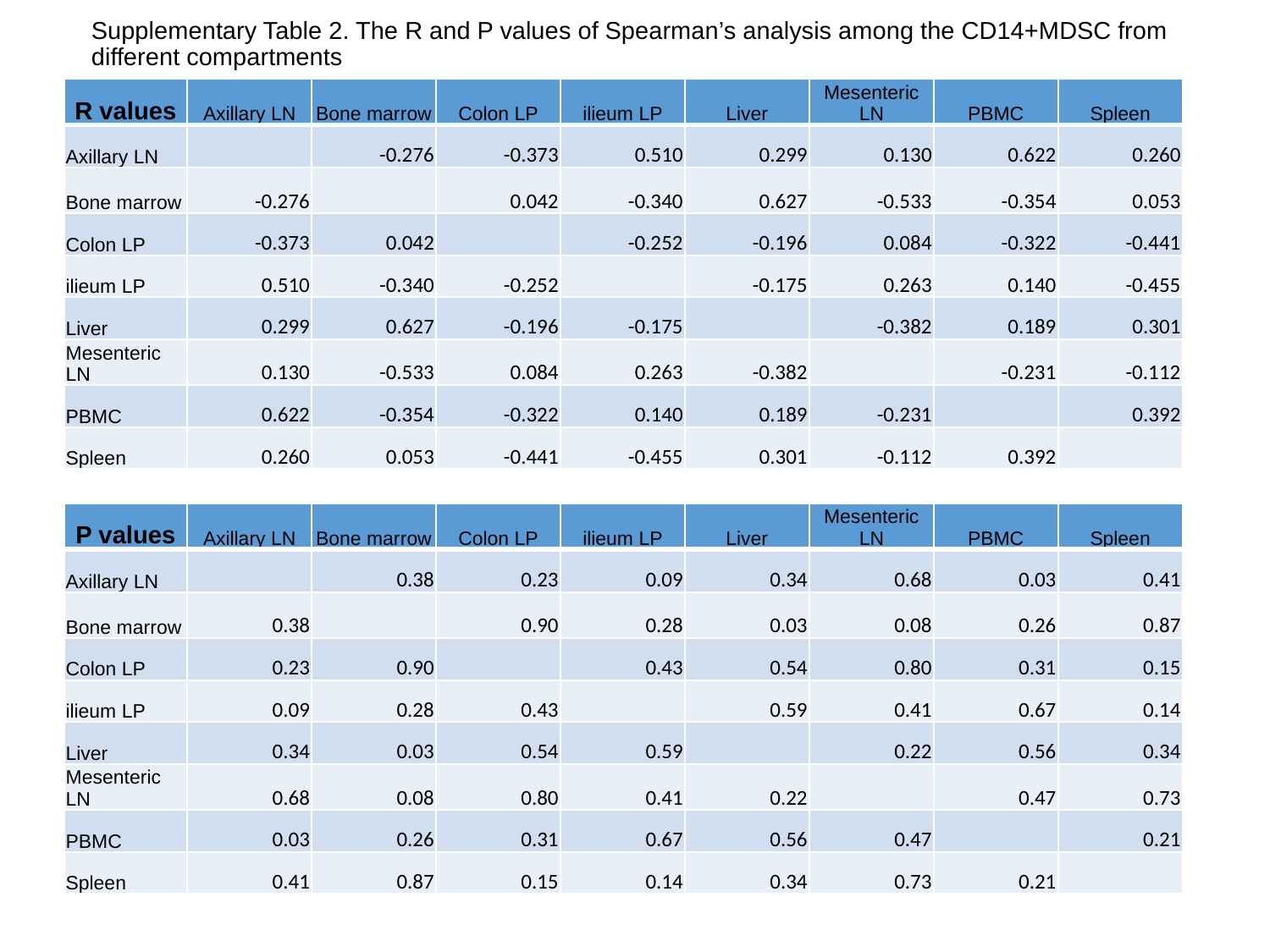

# Supplementary Table 2. The R and P values of Spearman’s analysis among the CD14+MDSC from different compartments
| R values | Axillary LN | Bone marrow | Colon LP | ilieum LP | Liver | Mesenteric LN | PBMC | Spleen |
| --- | --- | --- | --- | --- | --- | --- | --- | --- |
| Axillary LN | | -0.276 | -0.373 | 0.510 | 0.299 | 0.130 | 0.622 | 0.260 |
| Bone marrow | -0.276 | | 0.042 | -0.340 | 0.627 | -0.533 | -0.354 | 0.053 |
| Colon LP | -0.373 | 0.042 | | -0.252 | -0.196 | 0.084 | -0.322 | -0.441 |
| ilieum LP | 0.510 | -0.340 | -0.252 | | -0.175 | 0.263 | 0.140 | -0.455 |
| Liver | 0.299 | 0.627 | -0.196 | -0.175 | | -0.382 | 0.189 | 0.301 |
| Mesenteric LN | 0.130 | -0.533 | 0.084 | 0.263 | -0.382 | | -0.231 | -0.112 |
| PBMC | 0.622 | -0.354 | -0.322 | 0.140 | 0.189 | -0.231 | | 0.392 |
| Spleen | 0.260 | 0.053 | -0.441 | -0.455 | 0.301 | -0.112 | 0.392 | |
| P values | Axillary LN | Bone marrow | Colon LP | ilieum LP | Liver | Mesenteric LN | PBMC | Spleen |
| --- | --- | --- | --- | --- | --- | --- | --- | --- |
| Axillary LN | | 0.38 | 0.23 | 0.09 | 0.34 | 0.68 | 0.03 | 0.41 |
| Bone marrow | 0.38 | | 0.90 | 0.28 | 0.03 | 0.08 | 0.26 | 0.87 |
| Colon LP | 0.23 | 0.90 | | 0.43 | 0.54 | 0.80 | 0.31 | 0.15 |
| ilieum LP | 0.09 | 0.28 | 0.43 | | 0.59 | 0.41 | 0.67 | 0.14 |
| Liver | 0.34 | 0.03 | 0.54 | 0.59 | | 0.22 | 0.56 | 0.34 |
| Mesenteric LN | 0.68 | 0.08 | 0.80 | 0.41 | 0.22 | | 0.47 | 0.73 |
| PBMC | 0.03 | 0.26 | 0.31 | 0.67 | 0.56 | 0.47 | | 0.21 |
| Spleen | 0.41 | 0.87 | 0.15 | 0.14 | 0.34 | 0.73 | 0.21 | |
