# Supplementary material for: Paradoxical myeloid-derived suppressor cell reduction in the bone marrow of SIV chronically infected macaques
Source: PLoS Pathog. 2017 May 12;13(5):e1006395. doi: 10.1371/journal.ppat.1006395 (PMC5448820; doi:10.1371/journal.ppat.1006395)
Supplement: S3 Table — (PPTX) [file ppat.1006395.s013.pptx]

## Slide 1
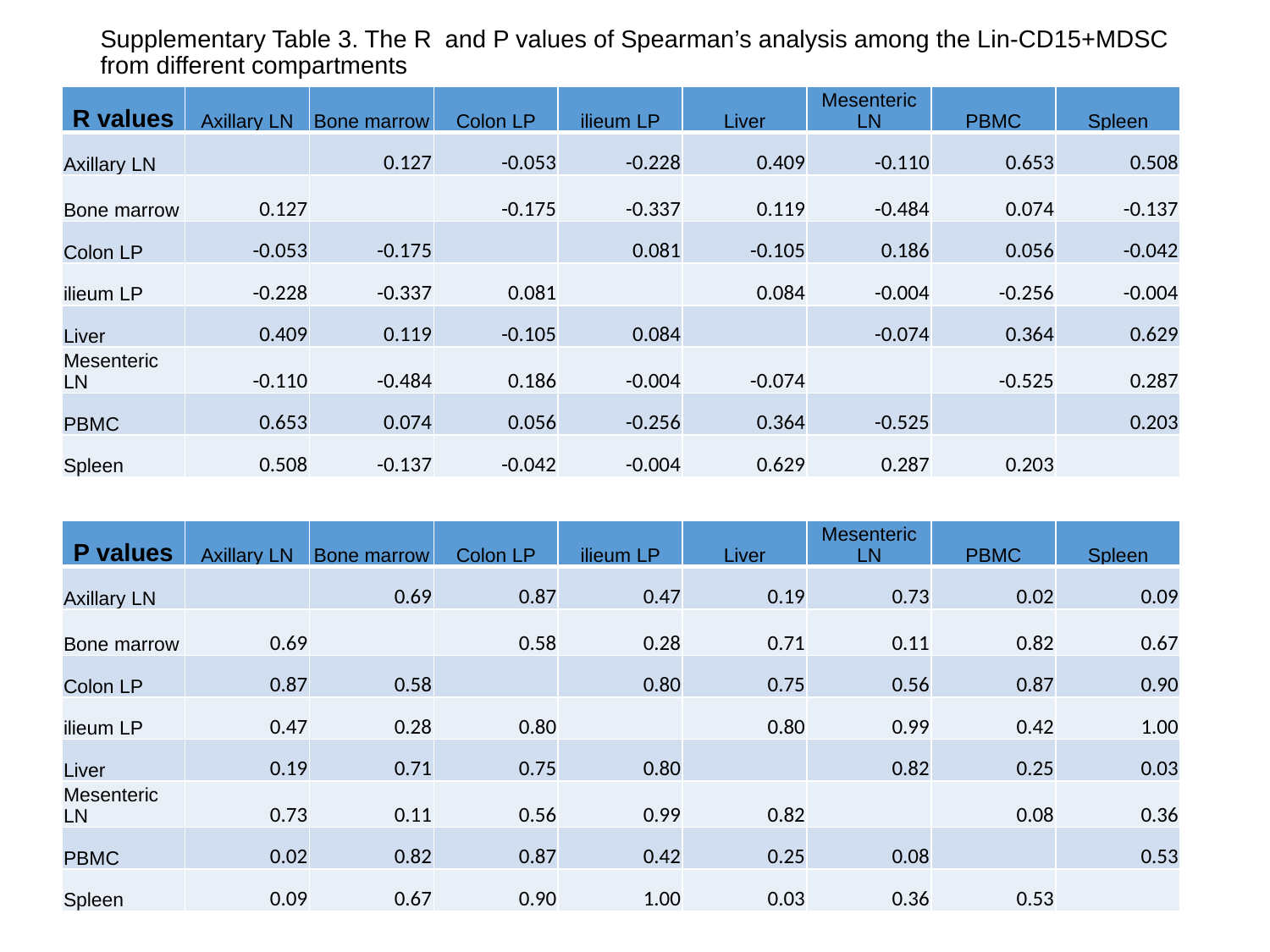

# Supplementary Table 3. The R and P values of Spearman’s analysis among the Lin-CD15+MDSC from different compartments
| R values | Axillary LN | Bone marrow | Colon LP | ilieum LP | Liver | Mesenteric LN | PBMC | Spleen |
| --- | --- | --- | --- | --- | --- | --- | --- | --- |
| Axillary LN | | 0.127 | -0.053 | -0.228 | 0.409 | -0.110 | 0.653 | 0.508 |
| Bone marrow | 0.127 | | -0.175 | -0.337 | 0.119 | -0.484 | 0.074 | -0.137 |
| Colon LP | -0.053 | -0.175 | | 0.081 | -0.105 | 0.186 | 0.056 | -0.042 |
| ilieum LP | -0.228 | -0.337 | 0.081 | | 0.084 | -0.004 | -0.256 | -0.004 |
| Liver | 0.409 | 0.119 | -0.105 | 0.084 | | -0.074 | 0.364 | 0.629 |
| Mesenteric LN | -0.110 | -0.484 | 0.186 | -0.004 | -0.074 | | -0.525 | 0.287 |
| PBMC | 0.653 | 0.074 | 0.056 | -0.256 | 0.364 | -0.525 | | 0.203 |
| Spleen | 0.508 | -0.137 | -0.042 | -0.004 | 0.629 | 0.287 | 0.203 | |
| P values | Axillary LN | Bone marrow | Colon LP | ilieum LP | Liver | Mesenteric LN | PBMC | Spleen |
| --- | --- | --- | --- | --- | --- | --- | --- | --- |
| Axillary LN | | 0.69 | 0.87 | 0.47 | 0.19 | 0.73 | 0.02 | 0.09 |
| Bone marrow | 0.69 | | 0.58 | 0.28 | 0.71 | 0.11 | 0.82 | 0.67 |
| Colon LP | 0.87 | 0.58 | | 0.80 | 0.75 | 0.56 | 0.87 | 0.90 |
| ilieum LP | 0.47 | 0.28 | 0.80 | | 0.80 | 0.99 | 0.42 | 1.00 |
| Liver | 0.19 | 0.71 | 0.75 | 0.80 | | 0.82 | 0.25 | 0.03 |
| Mesenteric LN | 0.73 | 0.11 | 0.56 | 0.99 | 0.82 | | 0.08 | 0.36 |
| PBMC | 0.02 | 0.82 | 0.87 | 0.42 | 0.25 | 0.08 | | 0.53 |
| Spleen | 0.09 | 0.67 | 0.90 | 1.00 | 0.03 | 0.36 | 0.53 | |
