# Supplementary material for: Paradoxical myeloid-derived suppressor cell reduction in the bone marrow of SIV chronically infected macaques
Source: PLoS Pathog. 2017 May 12;13(5):e1006395. doi: 10.1371/journal.ppat.1006395 (PMC5448820; doi:10.1371/journal.ppat.1006395)
Supplement: S4 Table — (PPTX) [file ppat.1006395.s014.pptx]

## Slide 1
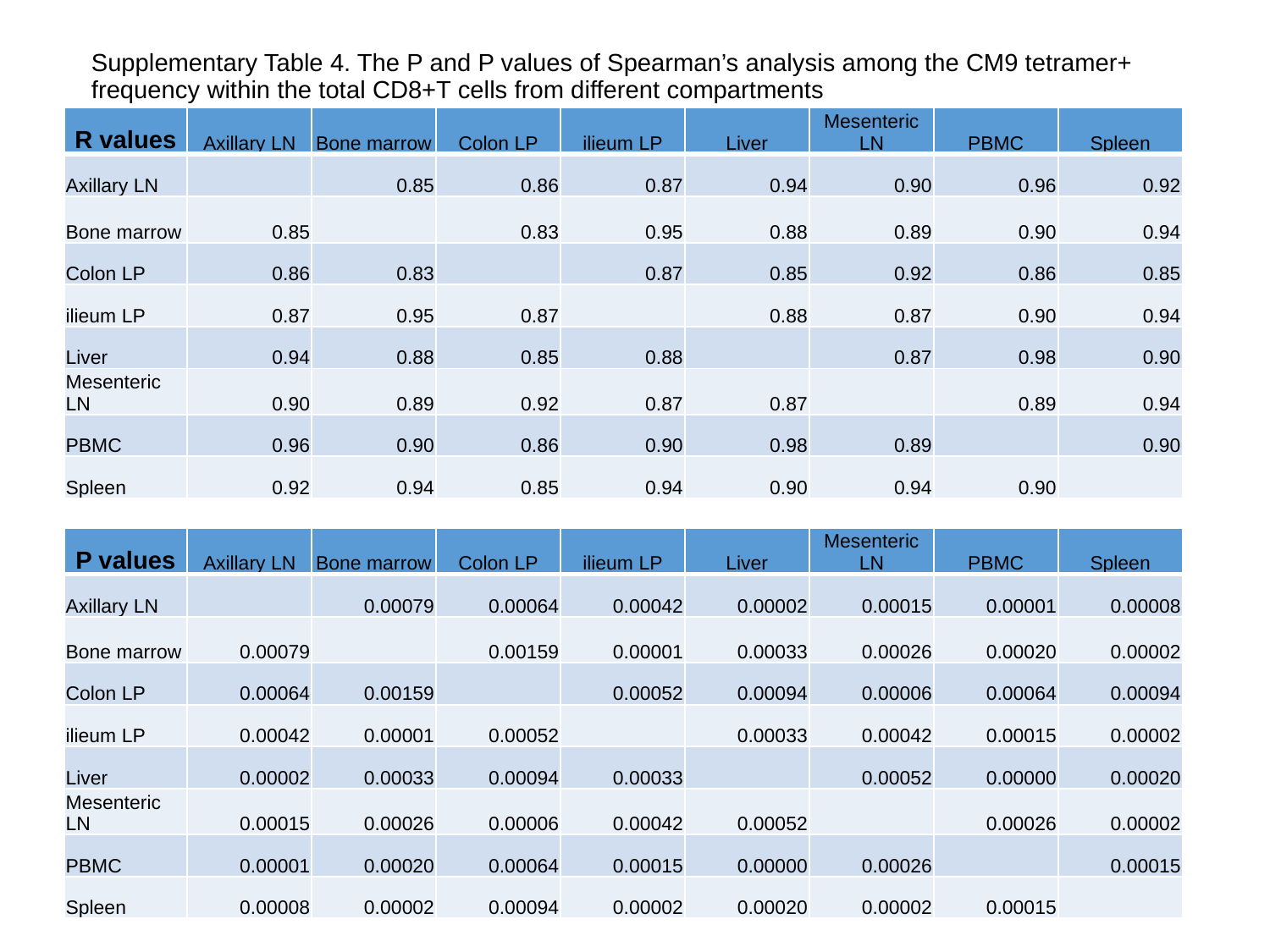

# Supplementary Table 4. The P and P values of Spearman’s analysis among the CM9 tetramer+ frequency within the total CD8+T cells from different compartments
| R values | Axillary LN | Bone marrow | Colon LP | ilieum LP | Liver | Mesenteric LN | PBMC | Spleen |
| --- | --- | --- | --- | --- | --- | --- | --- | --- |
| Axillary LN | | 0.85 | 0.86 | 0.87 | 0.94 | 0.90 | 0.96 | 0.92 |
| Bone marrow | 0.85 | | 0.83 | 0.95 | 0.88 | 0.89 | 0.90 | 0.94 |
| Colon LP | 0.86 | 0.83 | | 0.87 | 0.85 | 0.92 | 0.86 | 0.85 |
| ilieum LP | 0.87 | 0.95 | 0.87 | | 0.88 | 0.87 | 0.90 | 0.94 |
| Liver | 0.94 | 0.88 | 0.85 | 0.88 | | 0.87 | 0.98 | 0.90 |
| Mesenteric LN | 0.90 | 0.89 | 0.92 | 0.87 | 0.87 | | 0.89 | 0.94 |
| PBMC | 0.96 | 0.90 | 0.86 | 0.90 | 0.98 | 0.89 | | 0.90 |
| Spleen | 0.92 | 0.94 | 0.85 | 0.94 | 0.90 | 0.94 | 0.90 | |
| P values | Axillary LN | Bone marrow | Colon LP | ilieum LP | Liver | Mesenteric LN | PBMC | Spleen |
| --- | --- | --- | --- | --- | --- | --- | --- | --- |
| Axillary LN | | 0.00079 | 0.00064 | 0.00042 | 0.00002 | 0.00015 | 0.00001 | 0.00008 |
| Bone marrow | 0.00079 | | 0.00159 | 0.00001 | 0.00033 | 0.00026 | 0.00020 | 0.00002 |
| Colon LP | 0.00064 | 0.00159 | | 0.00052 | 0.00094 | 0.00006 | 0.00064 | 0.00094 |
| ilieum LP | 0.00042 | 0.00001 | 0.00052 | | 0.00033 | 0.00042 | 0.00015 | 0.00002 |
| Liver | 0.00002 | 0.00033 | 0.00094 | 0.00033 | | 0.00052 | 0.00000 | 0.00020 |
| Mesenteric LN | 0.00015 | 0.00026 | 0.00006 | 0.00042 | 0.00052 | | 0.00026 | 0.00002 |
| PBMC | 0.00001 | 0.00020 | 0.00064 | 0.00015 | 0.00000 | 0.00026 | | 0.00015 |
| Spleen | 0.00008 | 0.00002 | 0.00094 | 0.00002 | 0.00020 | 0.00002 | 0.00015 | |
