# Supplementary material for: Paradoxical myeloid-derived suppressor cell reduction in the bone marrow of SIV chronically infected macaques
Source: PLoS Pathog. 2017 May 12;13(5):e1006395. doi: 10.1371/journal.ppat.1006395 (PMC5448820; doi:10.1371/journal.ppat.1006395)
Supplement: S5 Table — (PPTX) [file ppat.1006395.s015.pptx]

## Slide 1
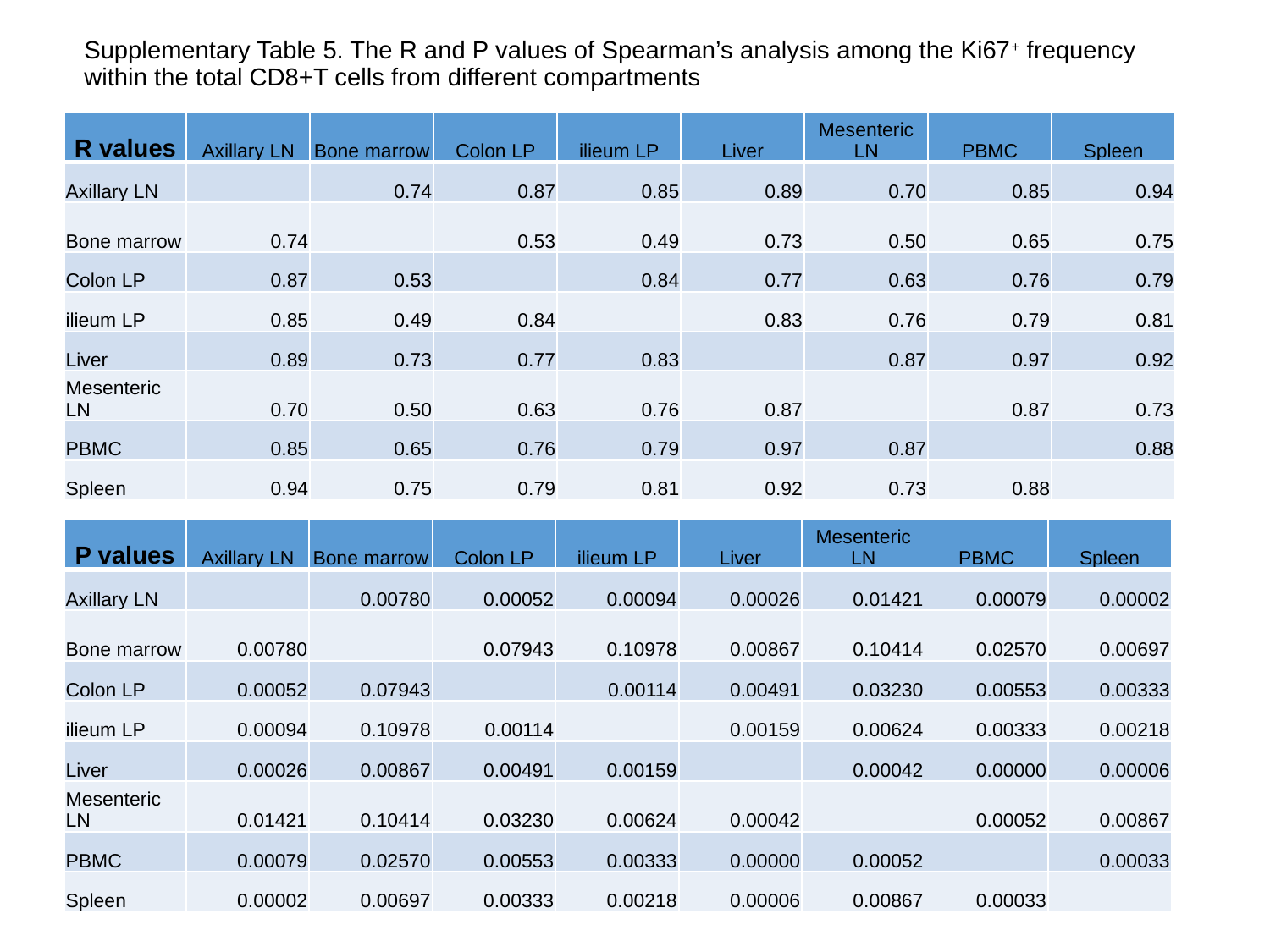

# Supplementary Table 5. The R and P values of Spearman’s analysis among the Ki67+ frequency within the total CD8+T cells from different compartments
| R values | Axillary LN | Bone marrow | Colon LP | ilieum LP | Liver | Mesenteric LN | PBMC | Spleen |
| --- | --- | --- | --- | --- | --- | --- | --- | --- |
| Axillary LN | | 0.74 | 0.87 | 0.85 | 0.89 | 0.70 | 0.85 | 0.94 |
| Bone marrow | 0.74 | | 0.53 | 0.49 | 0.73 | 0.50 | 0.65 | 0.75 |
| Colon LP | 0.87 | 0.53 | | 0.84 | 0.77 | 0.63 | 0.76 | 0.79 |
| ilieum LP | 0.85 | 0.49 | 0.84 | | 0.83 | 0.76 | 0.79 | 0.81 |
| Liver | 0.89 | 0.73 | 0.77 | 0.83 | | 0.87 | 0.97 | 0.92 |
| Mesenteric LN | 0.70 | 0.50 | 0.63 | 0.76 | 0.87 | | 0.87 | 0.73 |
| PBMC | 0.85 | 0.65 | 0.76 | 0.79 | 0.97 | 0.87 | | 0.88 |
| Spleen | 0.94 | 0.75 | 0.79 | 0.81 | 0.92 | 0.73 | 0.88 | |
| P values | Axillary LN | Bone marrow | Colon LP | ilieum LP | Liver | Mesenteric LN | PBMC | Spleen |
| --- | --- | --- | --- | --- | --- | --- | --- | --- |
| Axillary LN | | 0.00780 | 0.00052 | 0.00094 | 0.00026 | 0.01421 | 0.00079 | 0.00002 |
| Bone marrow | 0.00780 | | 0.07943 | 0.10978 | 0.00867 | 0.10414 | 0.02570 | 0.00697 |
| Colon LP | 0.00052 | 0.07943 | | 0.00114 | 0.00491 | 0.03230 | 0.00553 | 0.00333 |
| ilieum LP | 0.00094 | 0.10978 | 0.00114 | | 0.00159 | 0.00624 | 0.00333 | 0.00218 |
| Liver | 0.00026 | 0.00867 | 0.00491 | 0.00159 | | 0.00042 | 0.00000 | 0.00006 |
| Mesenteric LN | 0.01421 | 0.10414 | 0.03230 | 0.00624 | 0.00042 | | 0.00052 | 0.00867 |
| PBMC | 0.00079 | 0.02570 | 0.00553 | 0.00333 | 0.00000 | 0.00052 | | 0.00033 |
| Spleen | 0.00002 | 0.00697 | 0.00333 | 0.00218 | 0.00006 | 0.00867 | 0.00033 | |
